# Supplementary material for: Synthesis, Antimicrobial Activity, and Tyrosinase Inhibition by Multifunctional 3,4-Dihydroxy-Phenyl Peptidomimetics
Source: Int J Mol Sci. 2025 Feb 17;26(4):1702. doi: 10.3390/ijms26041702 (PMC11855086; doi:10.3390/ijms26041702)
Supplement: Supplementary file 1 [file ijms-26-01702-s001.zip › ijms-3391989-supplementary.pdf]

# Supporting Information

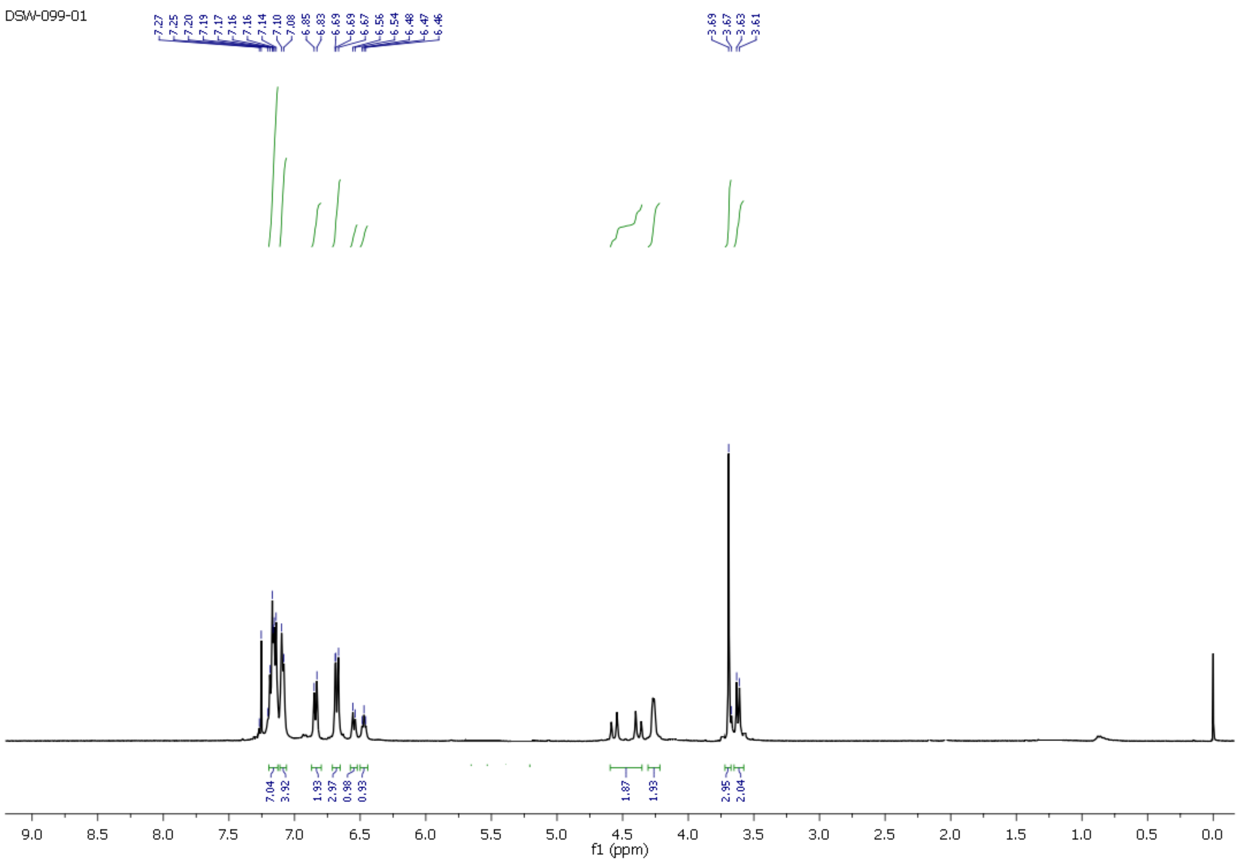

**Figure S1.** $^{1}H$ NMR (400 MHz, $CDCl_3$) spectra of compound **5a**

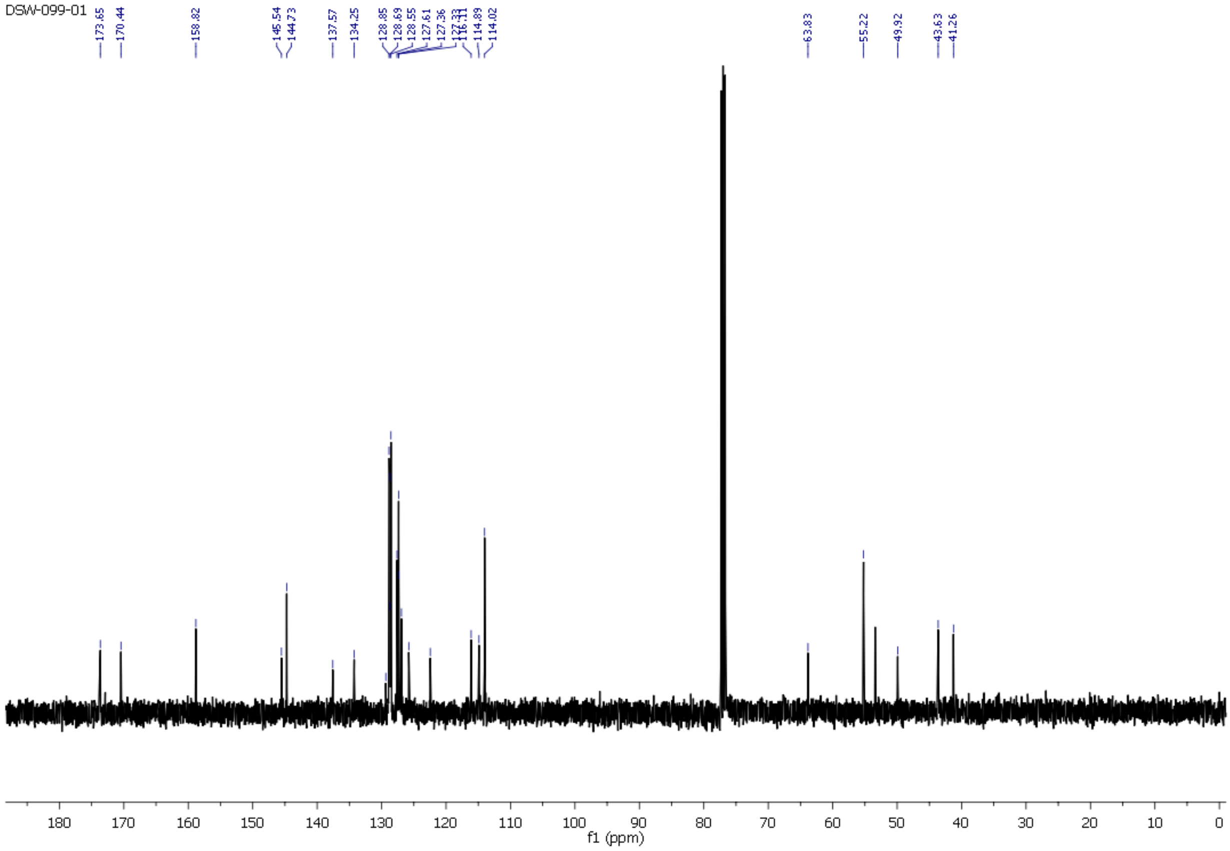


**Figure S2.** $^{13}C$ NMR (100 MHz, $CDCl_3$) spectra of compound **5a**

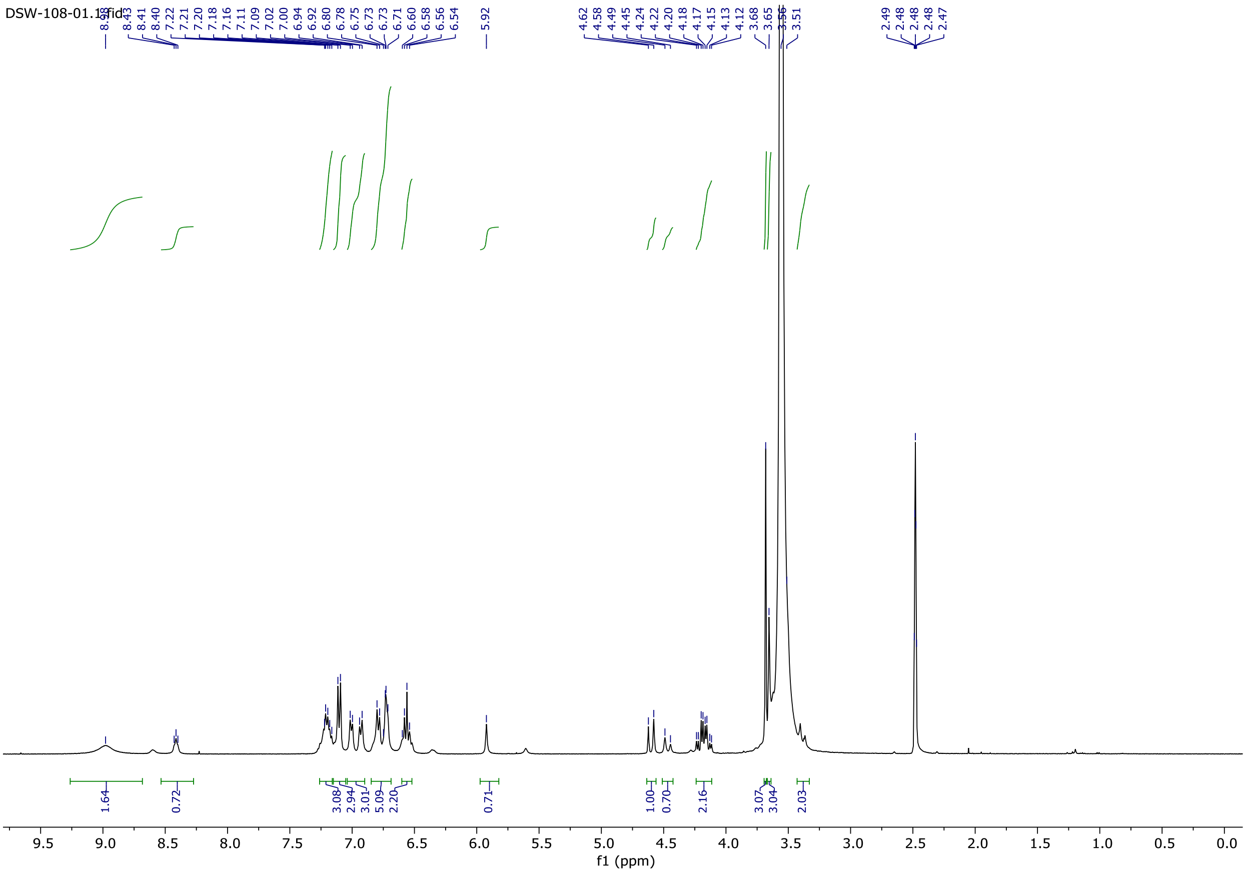


**Figure S3.** $^1H$ NMR (400 MHz, DMSO) spectra of compound 5b

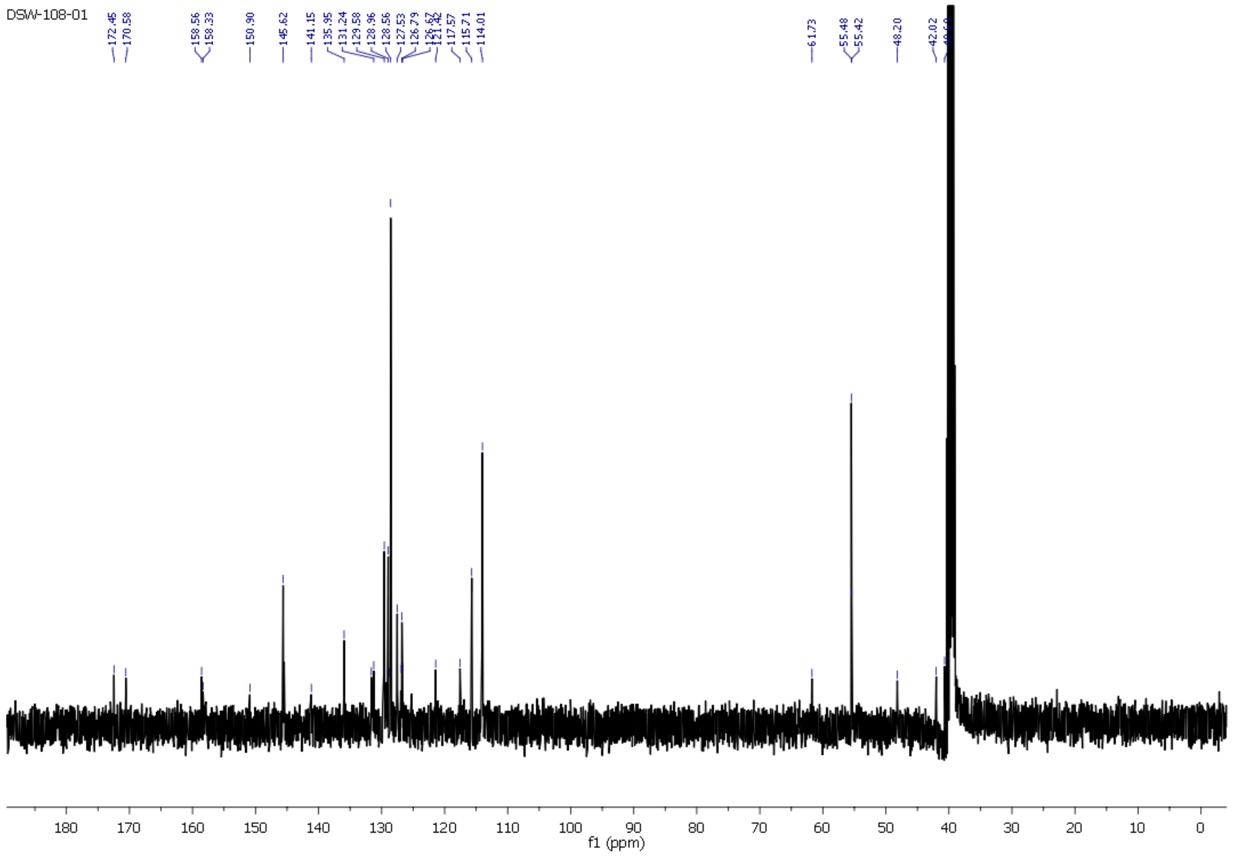

**Figure S4.** $^{13}C$ NMR (100 MHz, DMSO) spectra of compound **5b**

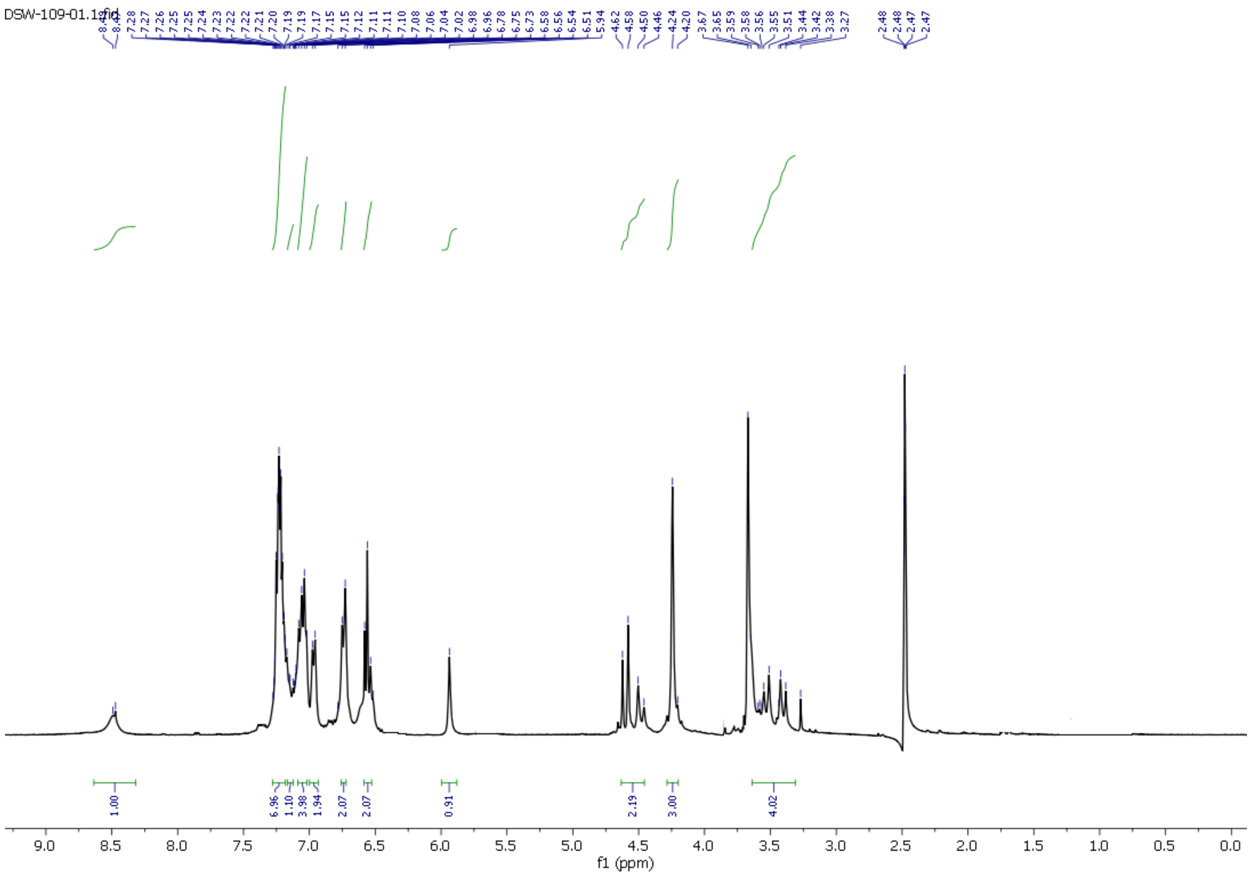

**Figure S5.** $^{1}H$ NMR (400 MHz, DMSO) spectra of compound **5c**

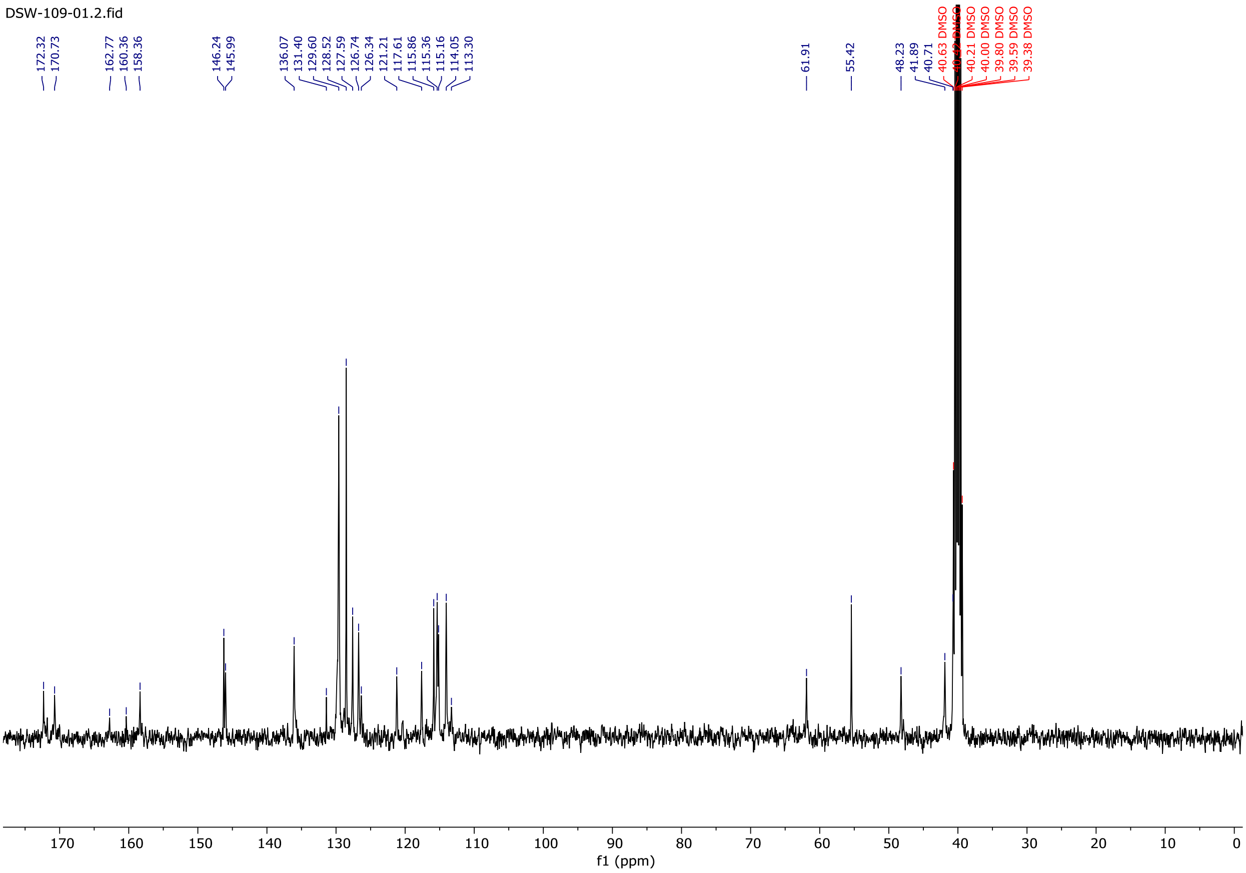


**Figure S6.** $^{13}C$ NMR (100 MHz, DMSO) spectra of compound **5c**

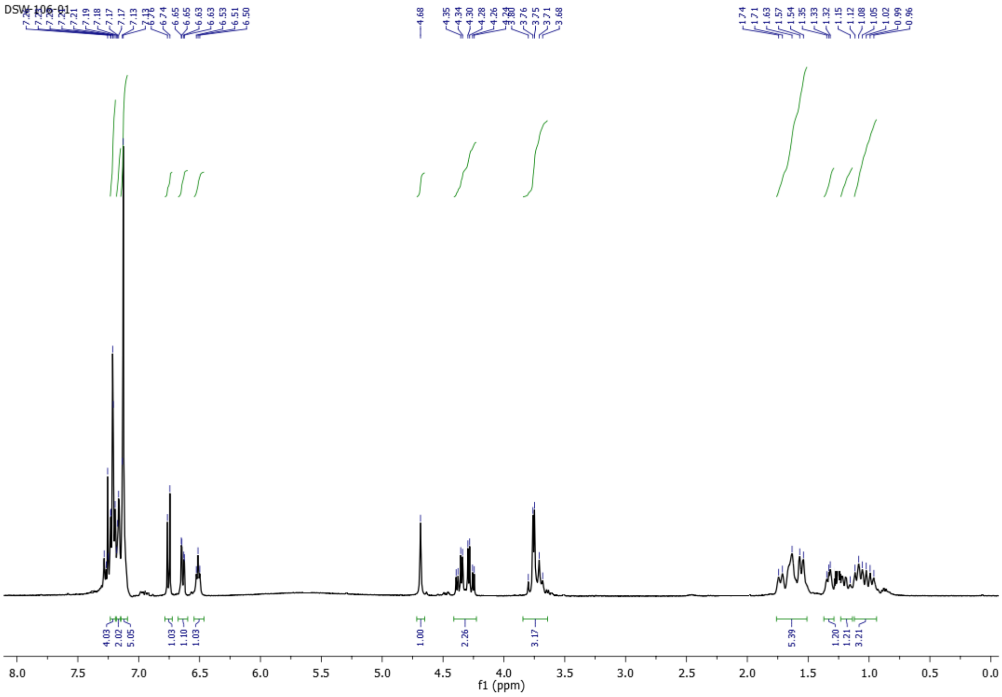


**Figure S7.** $^{1}H$ NMR (400 MHz, $CDCl_3$) spectra of compound **5d**

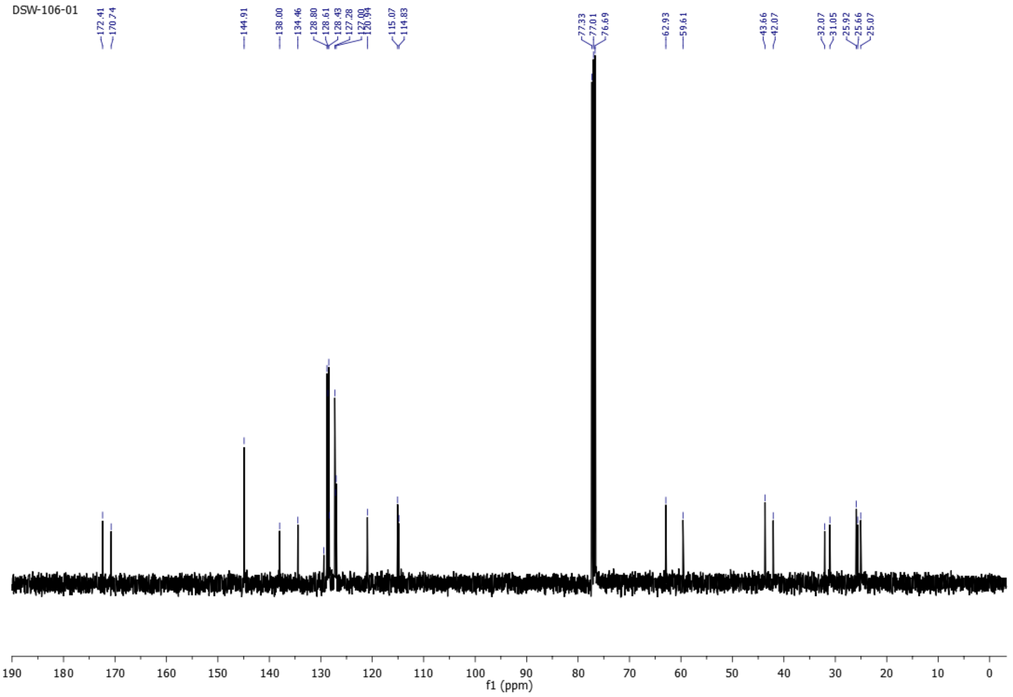


**Figure S8.** $^{13}C$ NMR (100 MHz, $CDCl_3$) spectra of compound **5d**

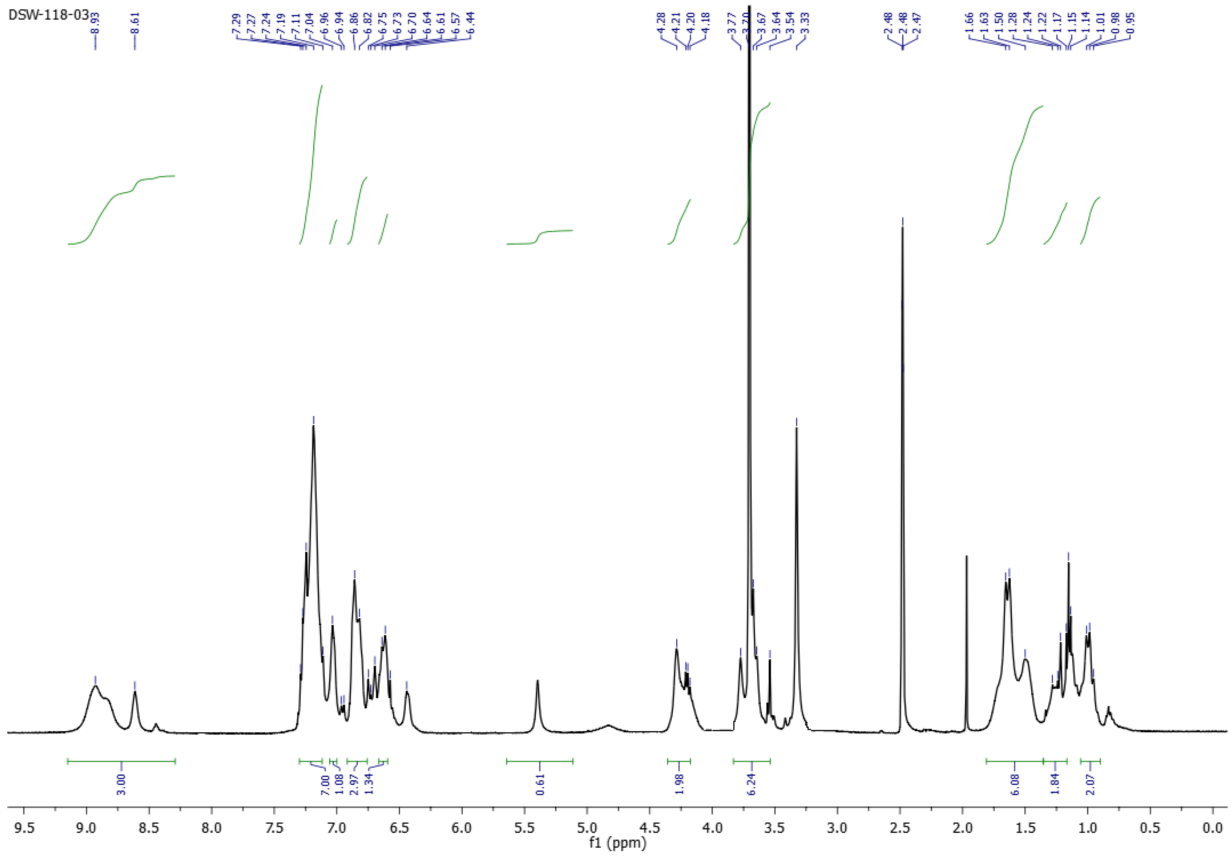


**Figure S9.** $^{1}H$ NMR (400 MHz, DMSO) spectra of compound 5e

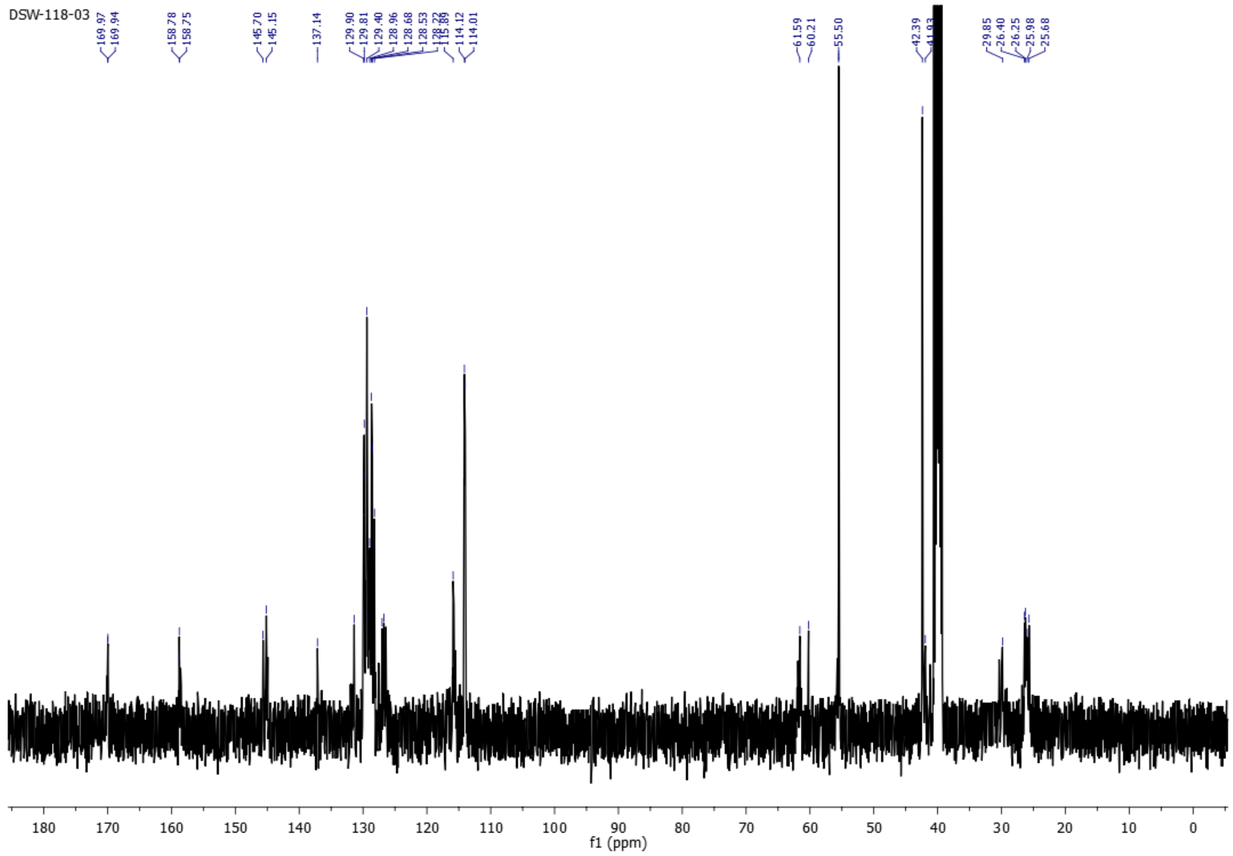


**Figure S10.** $^{13}C$ NMR (100 MHz, DMSO) spectra of compound **5e**

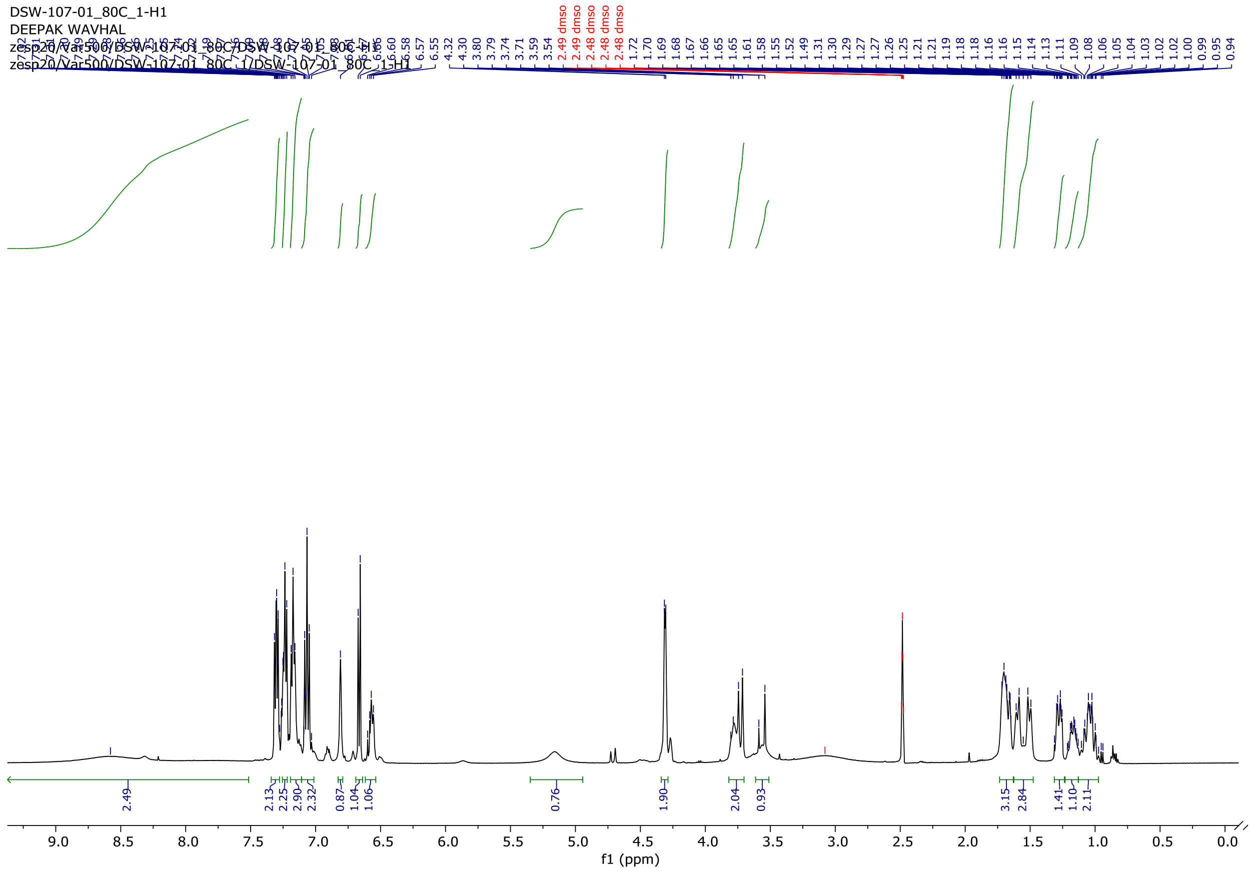


**Figure S11.** $^{1}H$ NMR (400 MHz, DMSO) spectra of compound **5f**

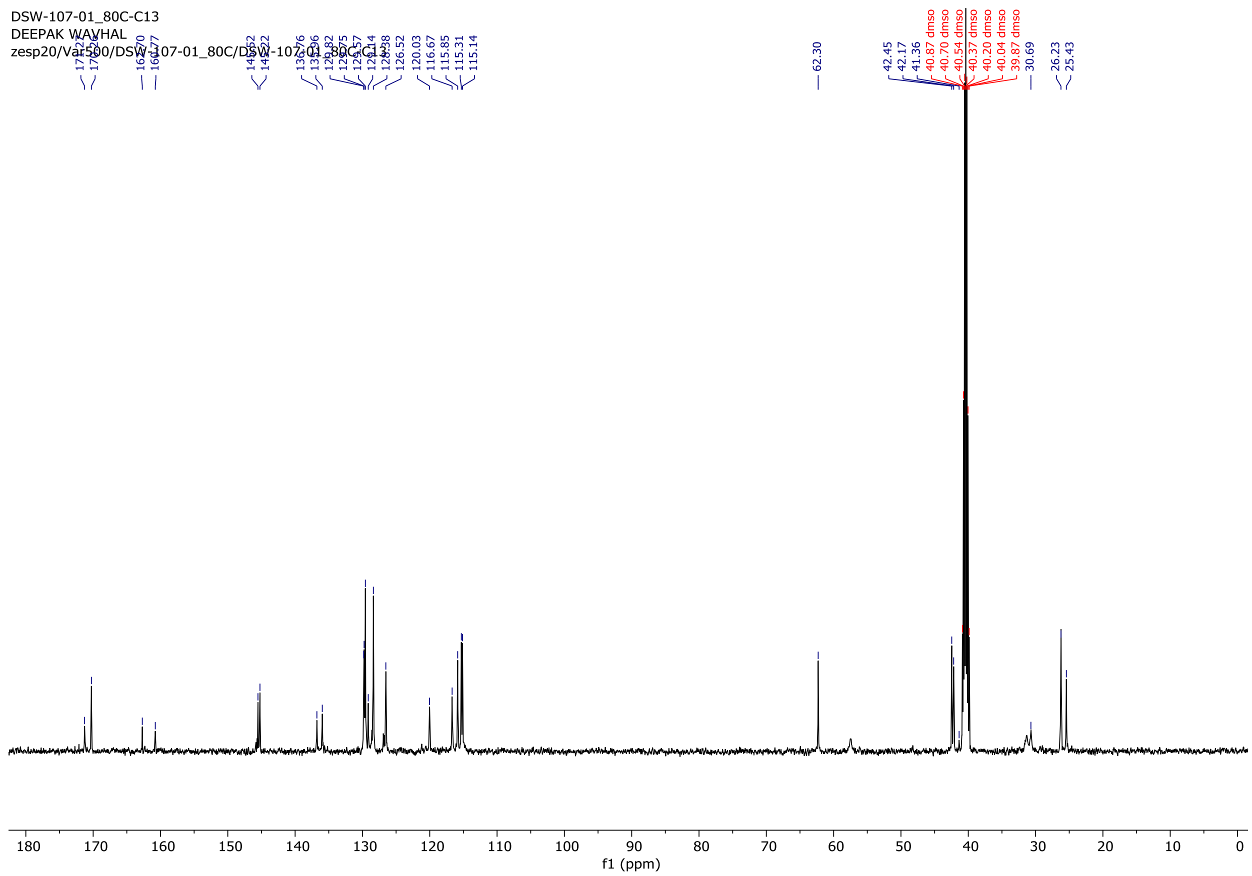

**Figure S12.** $^{13}$C NMR (100 MHz, DMSO) spectra of compound **5f**

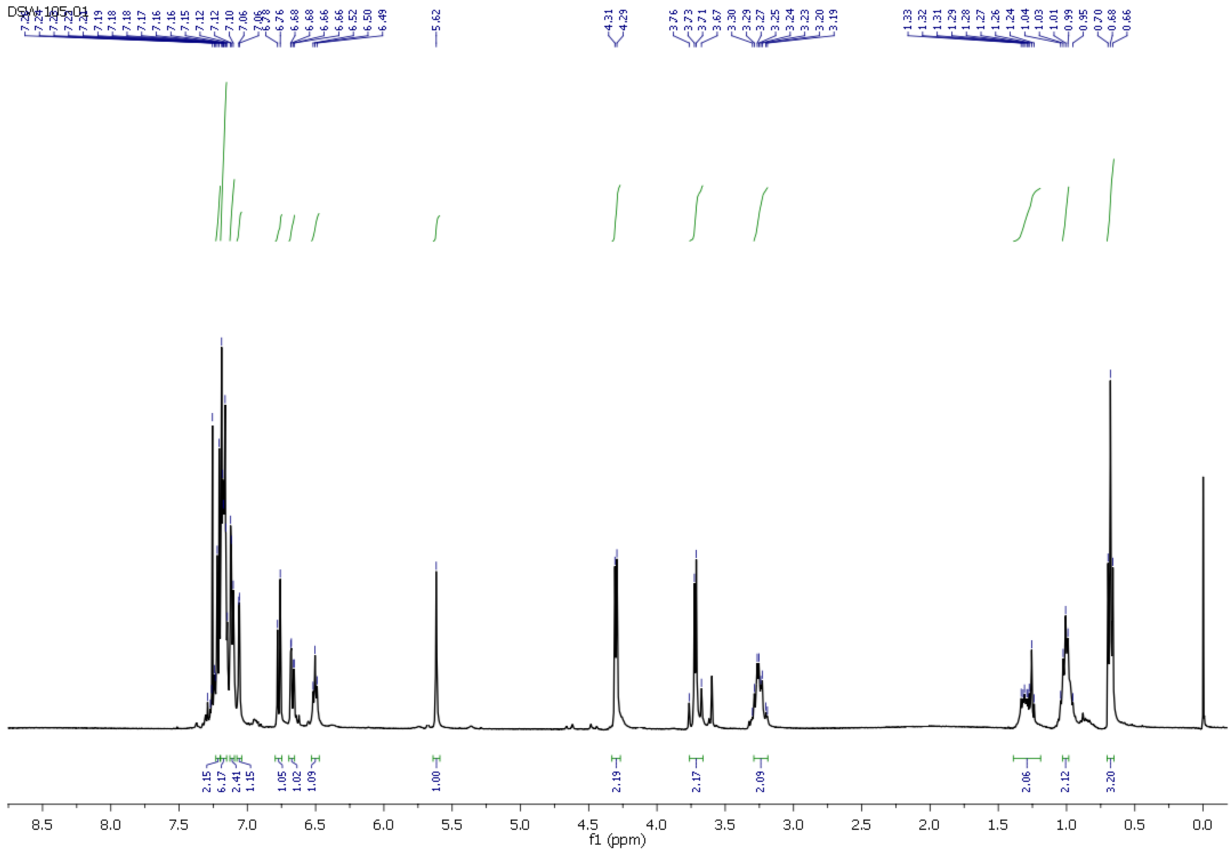

**Figure S13.** $^{1}$H NMR (400 MHz, $CDCl_3$) spectra of compound **5g**

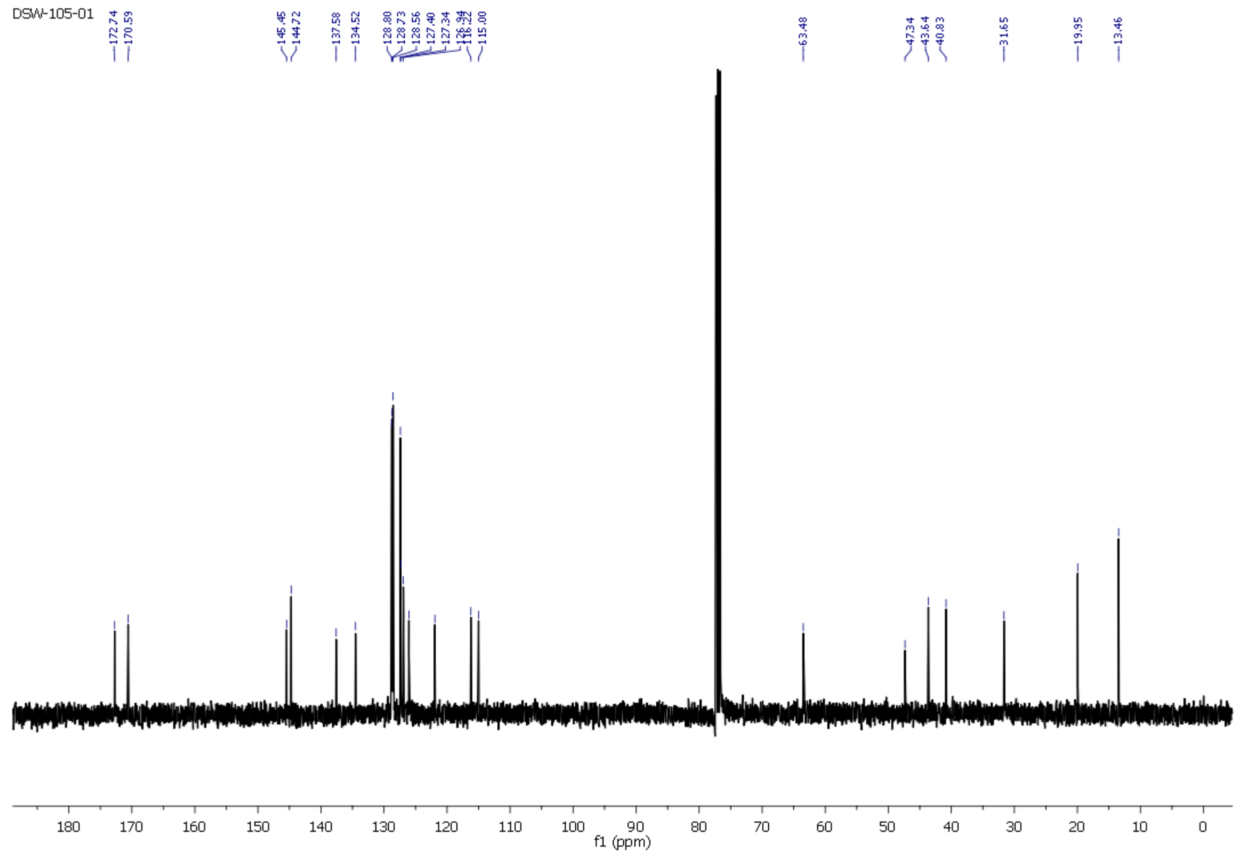


**Figure S14.** $^{13}$C NMR (400 MHz, $CDCl_3$) spectra of compound **5g**

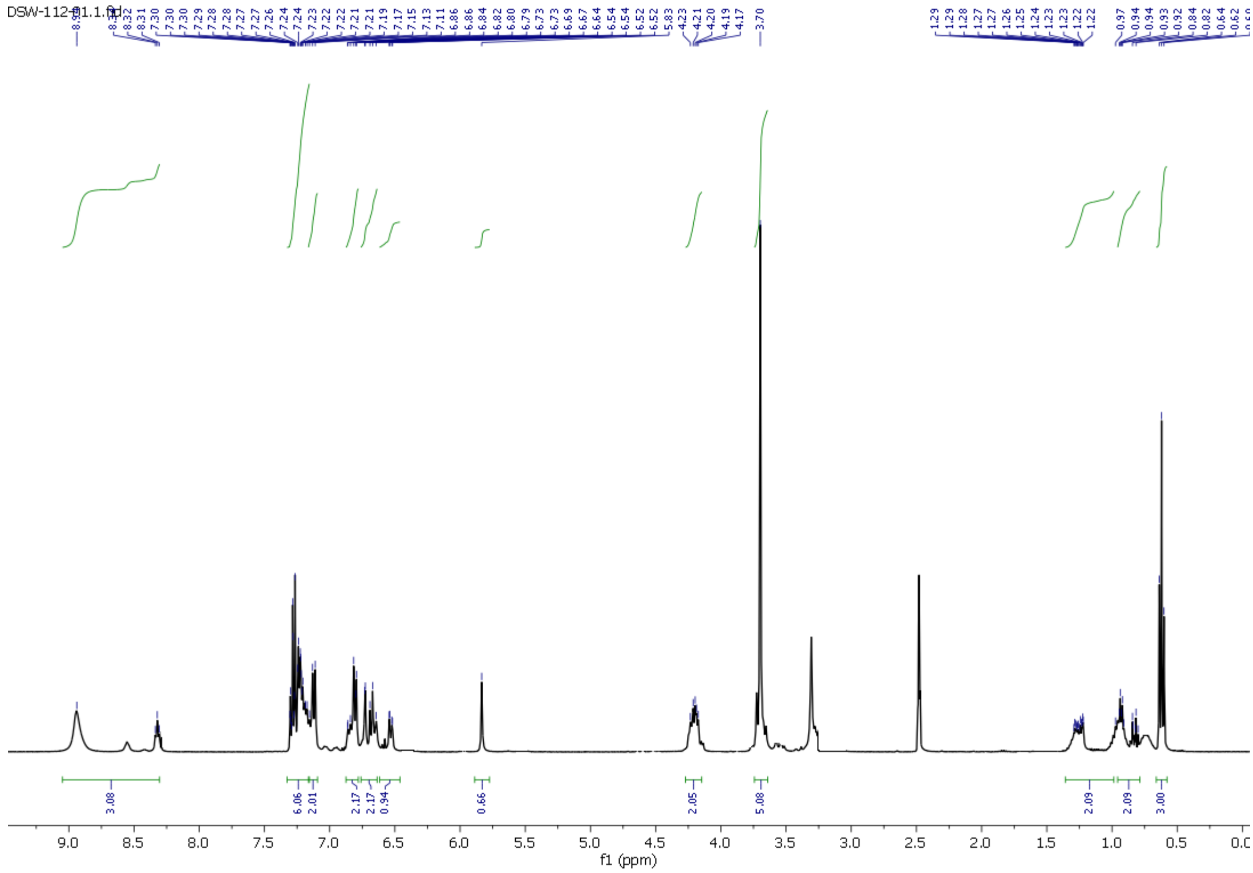


**Figure S15.** $^{1}$H NMR (400 MHz, DMSO) spectra of compound **5h**

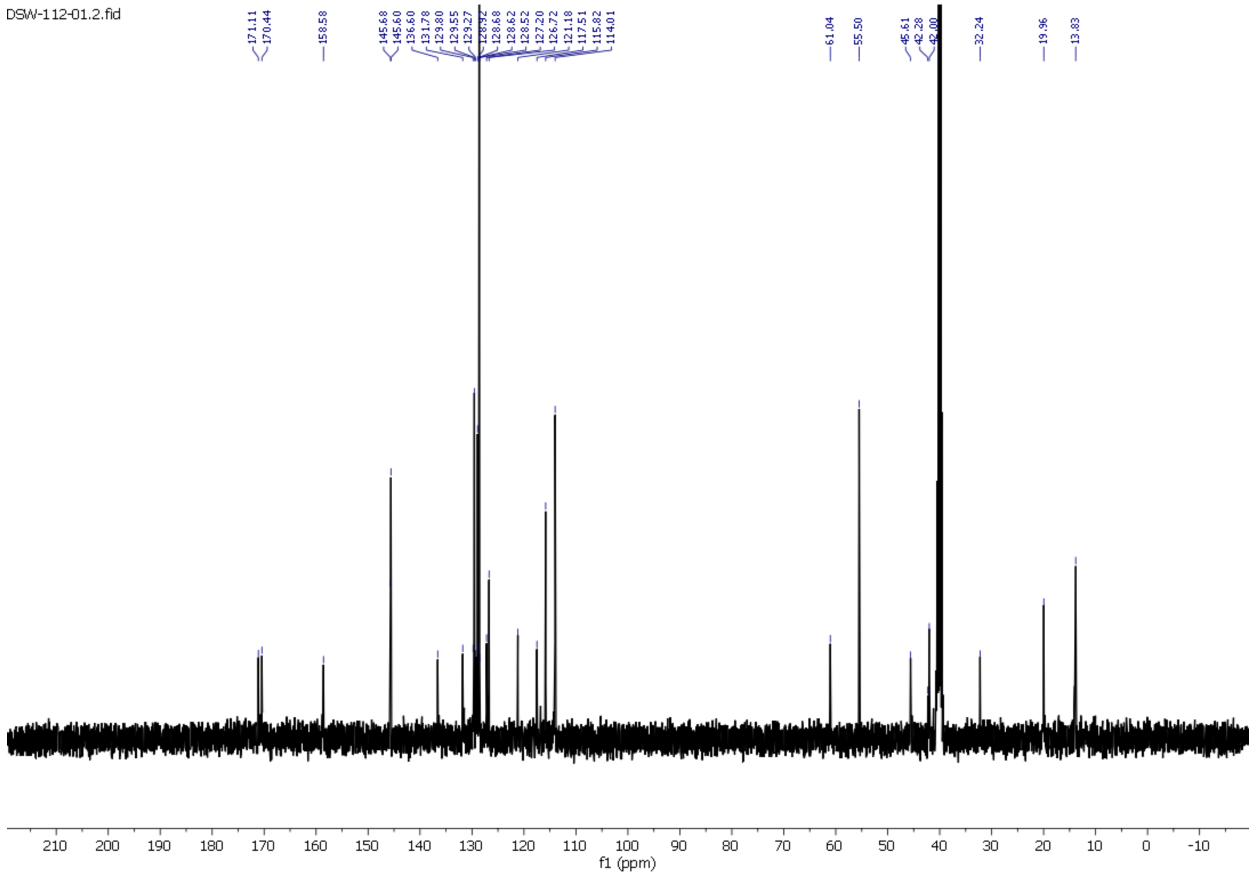

**Figure S16.** $^{13}C$ NMR (100 MHz, DMSO) spectra of compound **5h**

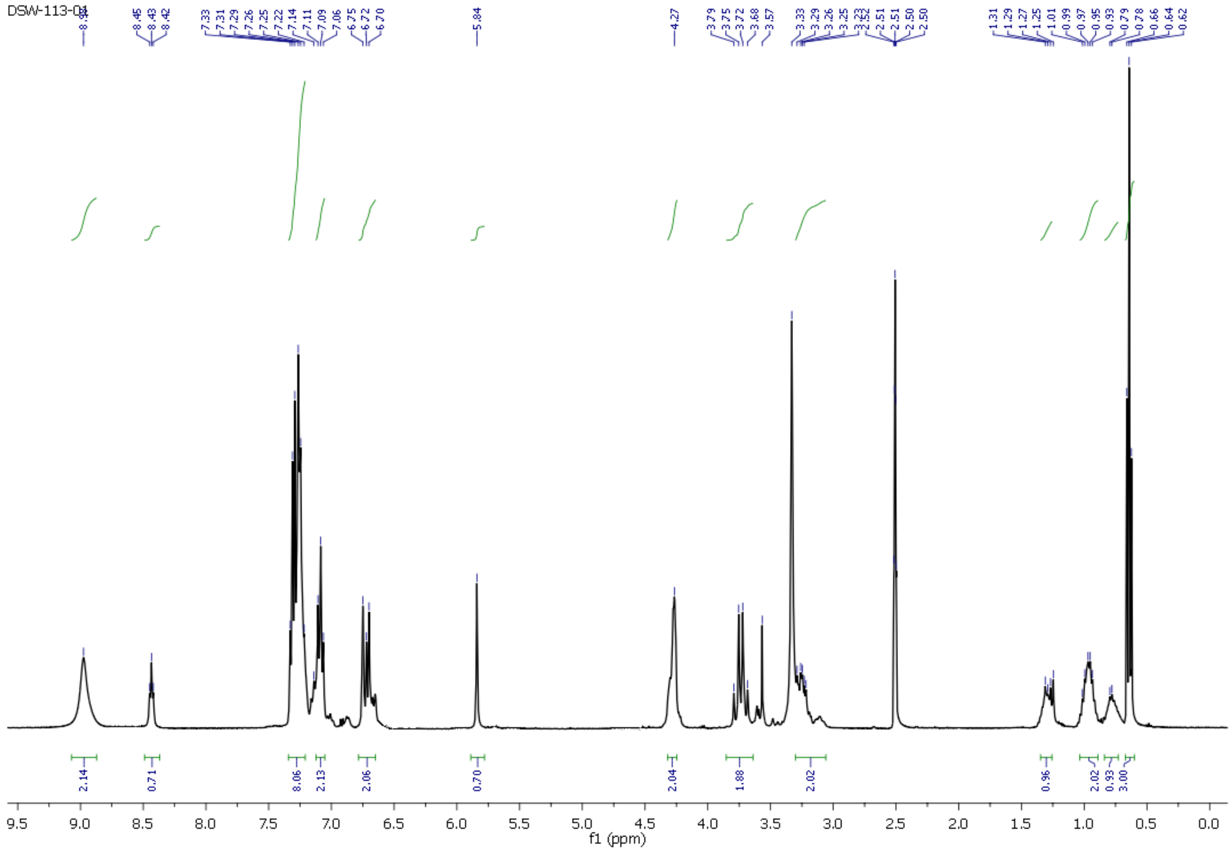

**Figure S17.** $^{1}H$ NMR (400 MHz, DMSO) spectra of compound 5i

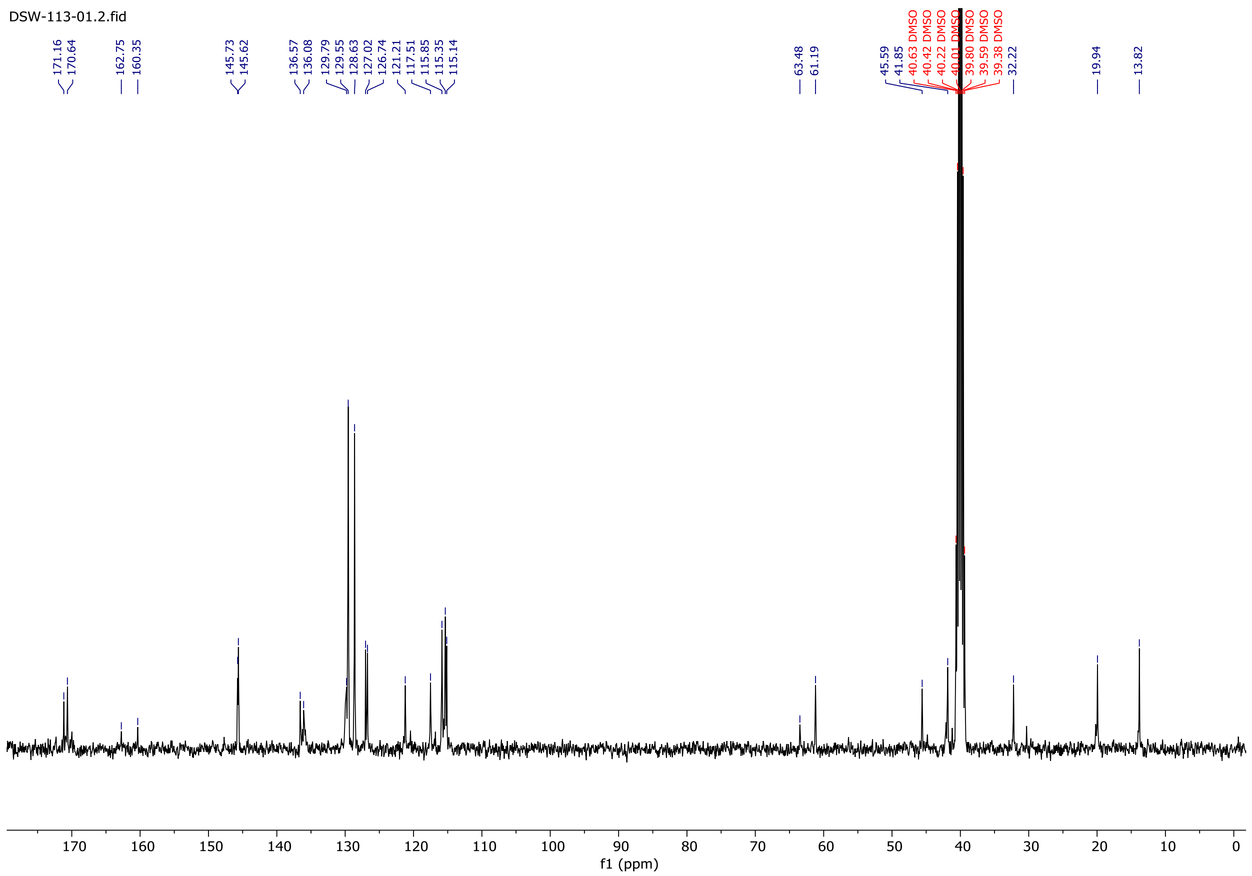

**Figure S18.** $^{13}C$ NMR (100 MHz, DMSO) spectra of compound **5i**

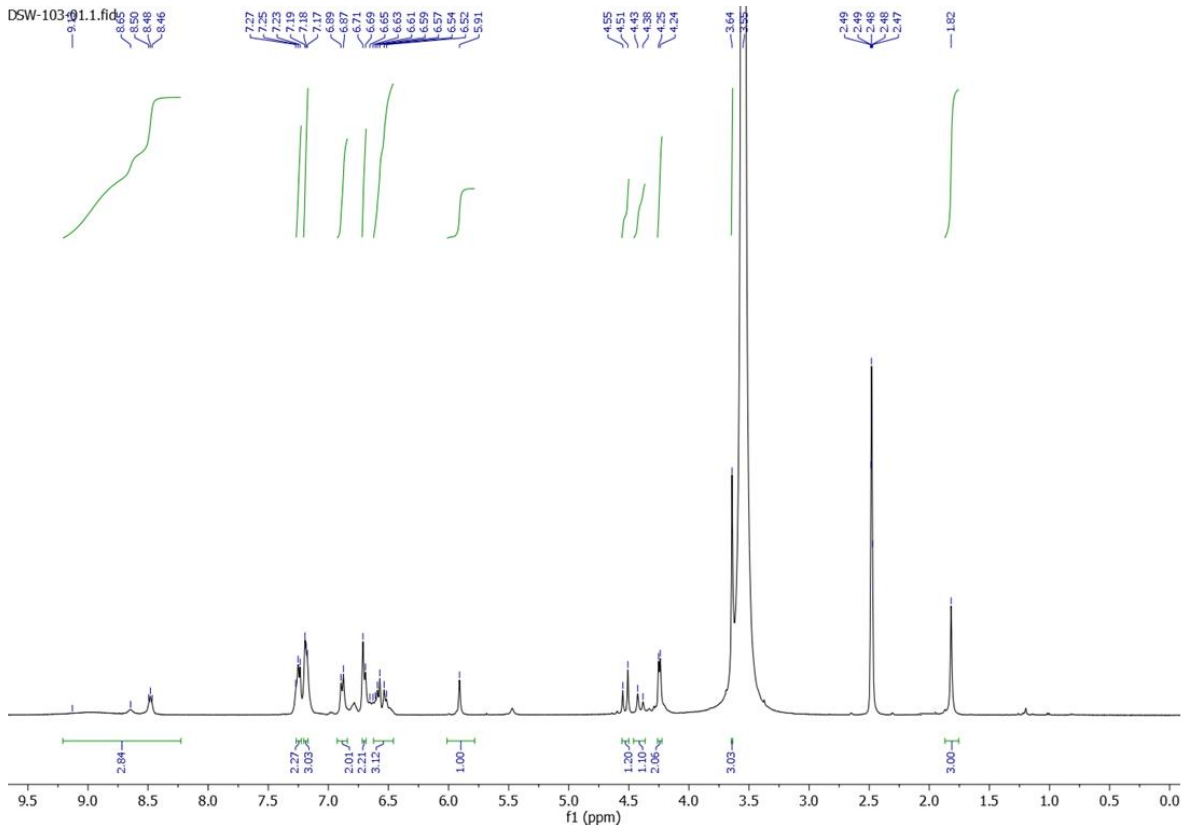

**Figure S19.** $^{1}H$ NMR (400 MHz, DMSO) spectra of compound **5j**

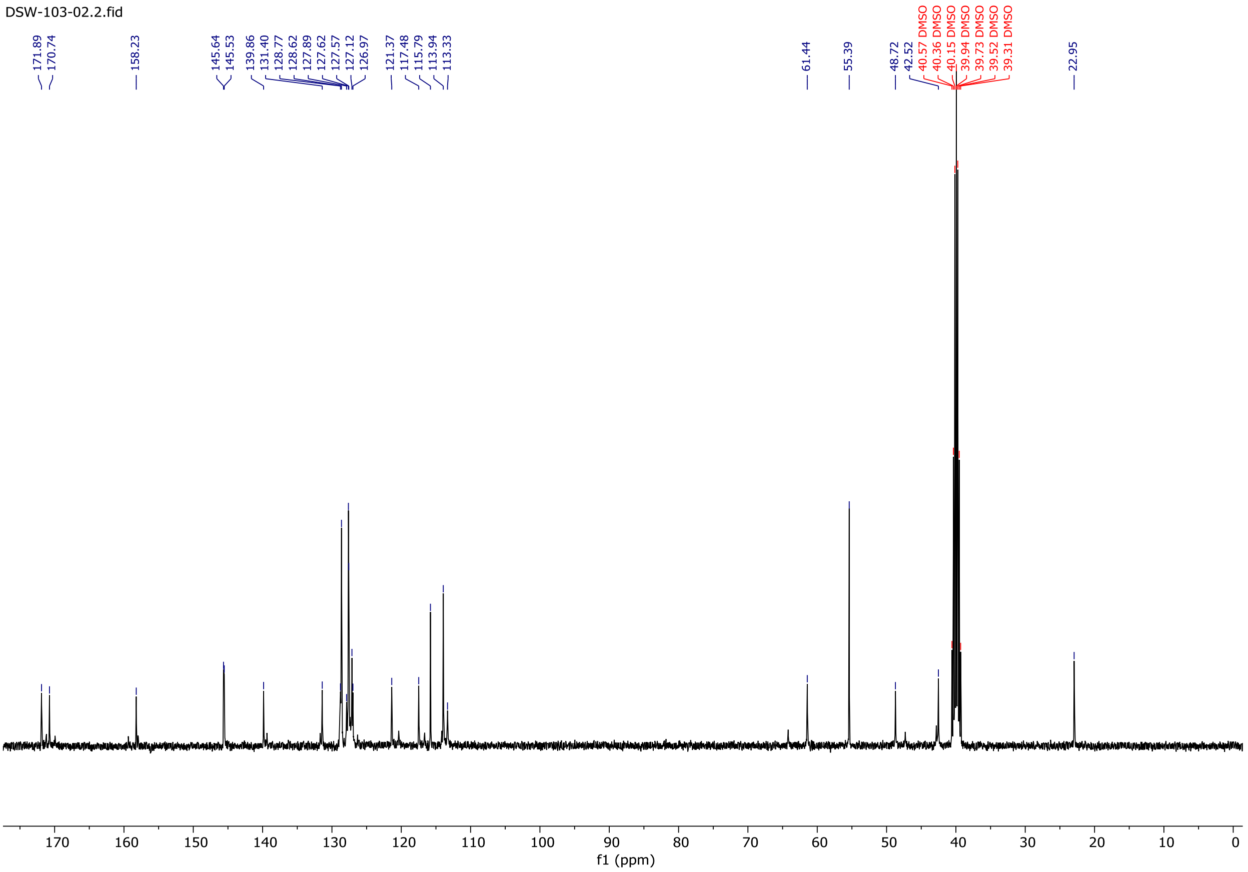

**Figure S20.** $^{13}C$ NMR (100 MHz, DMSO) spectra of compound **5j**

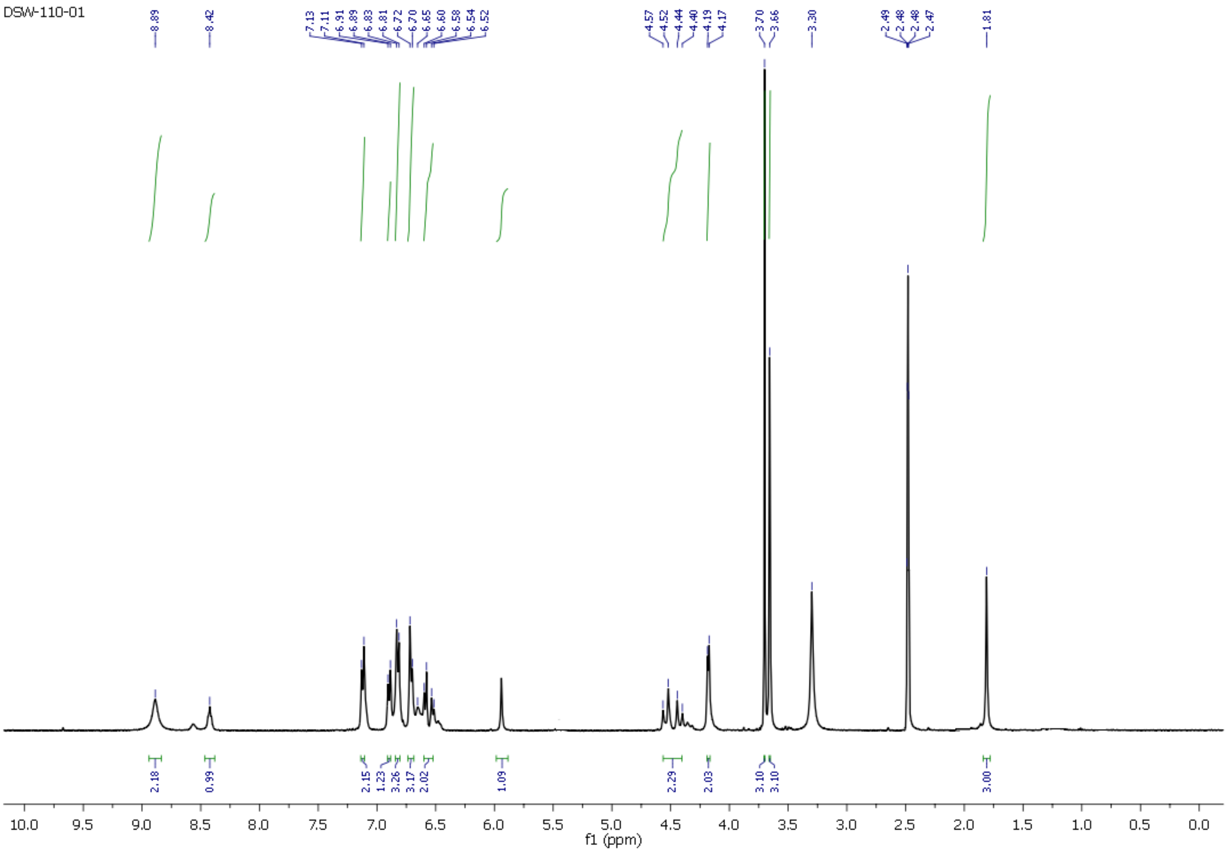

**Figure S21.** $^{1}H$ NMR (400 MHz, DMSO) spectra of compound **5k**

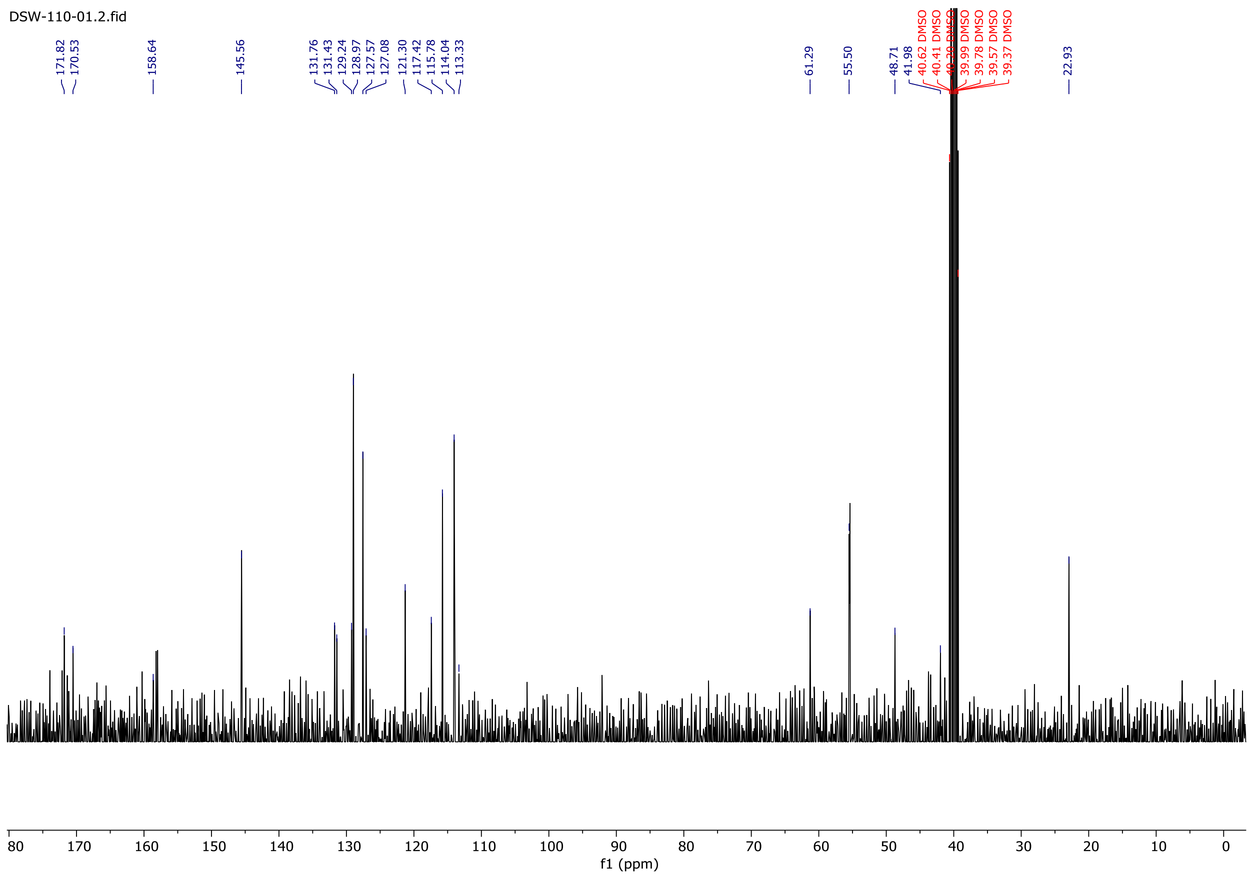

**Figure S22.** $^{13}$C CMR (100 MHz, DMSO) spectra of compound **5k**

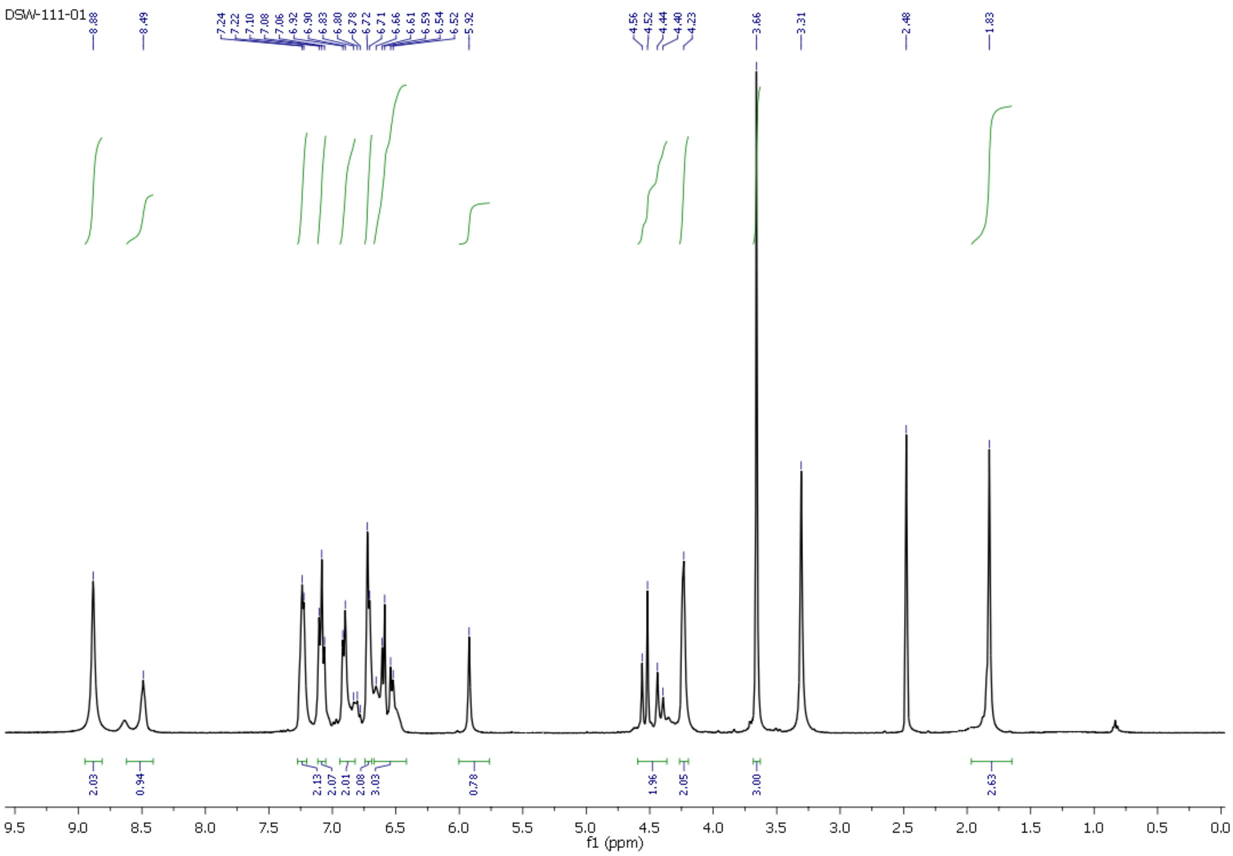

**Figure S23.** $^{1}$H NMR (400 MHz, DMSO) spectra of compound 5l

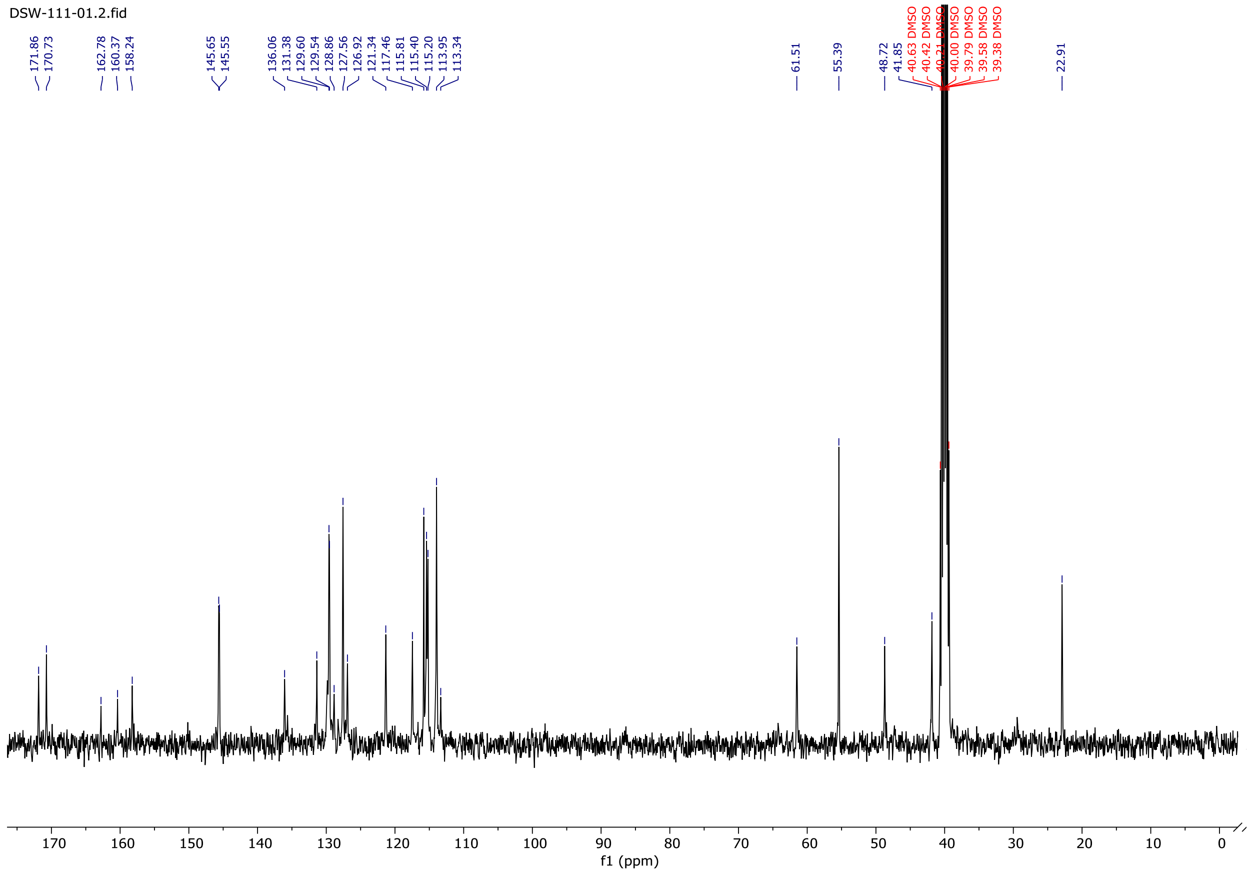

**Figure S24.** $^{13}$C NMR (100 MHz, DMSO) spectra of compound **5l**

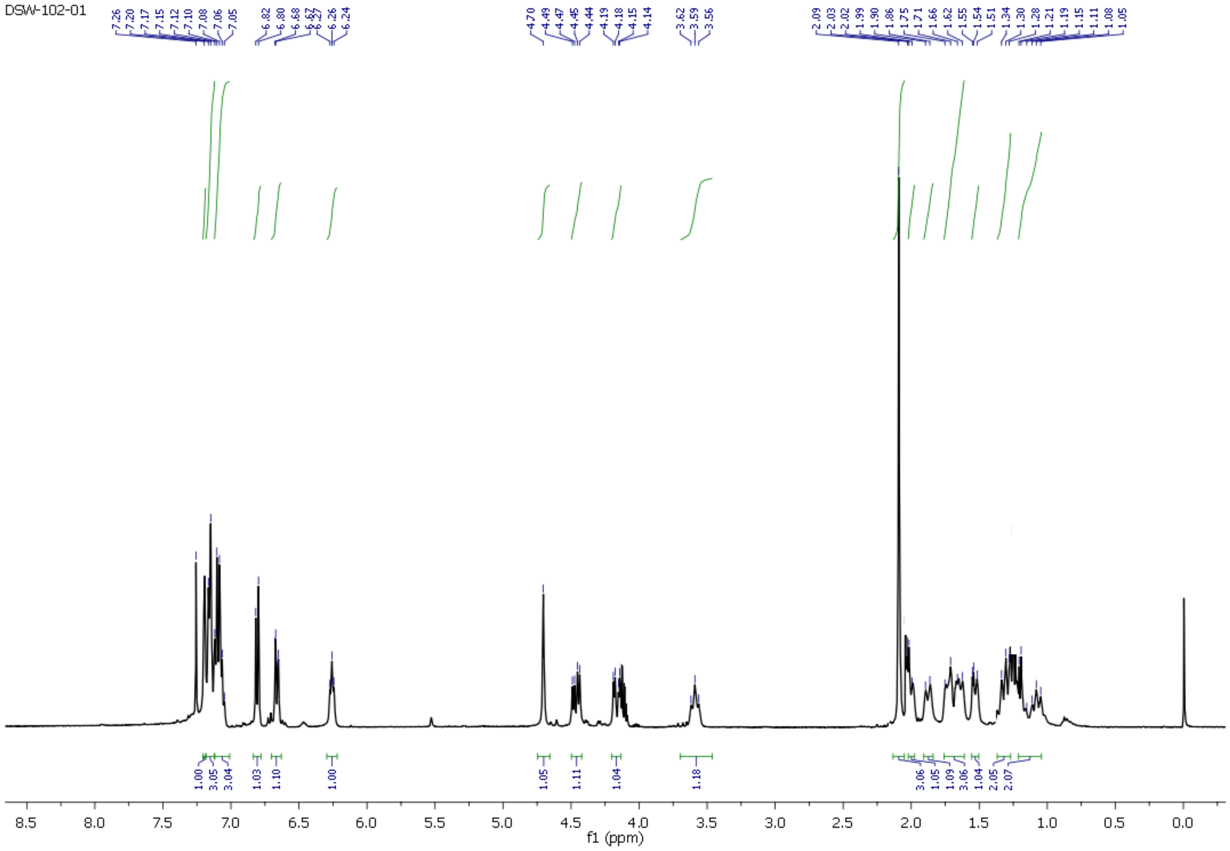

**Figure S25.** $^{1}$H NMR (400 MHz, DMSO) spectra of compound **5m**

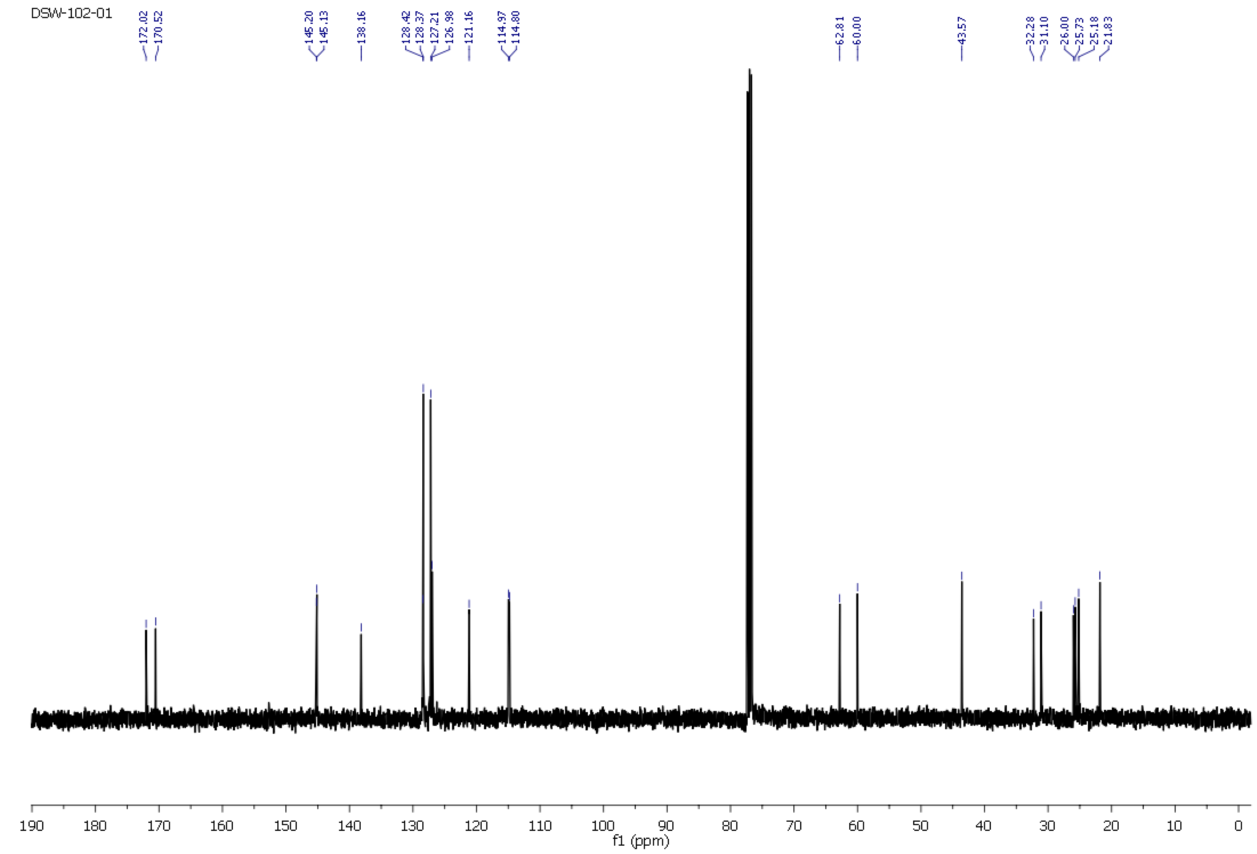


**Figure S26.** $^{13}C$ NMR (100 MHz, DMSO) spectra of compound **5m**

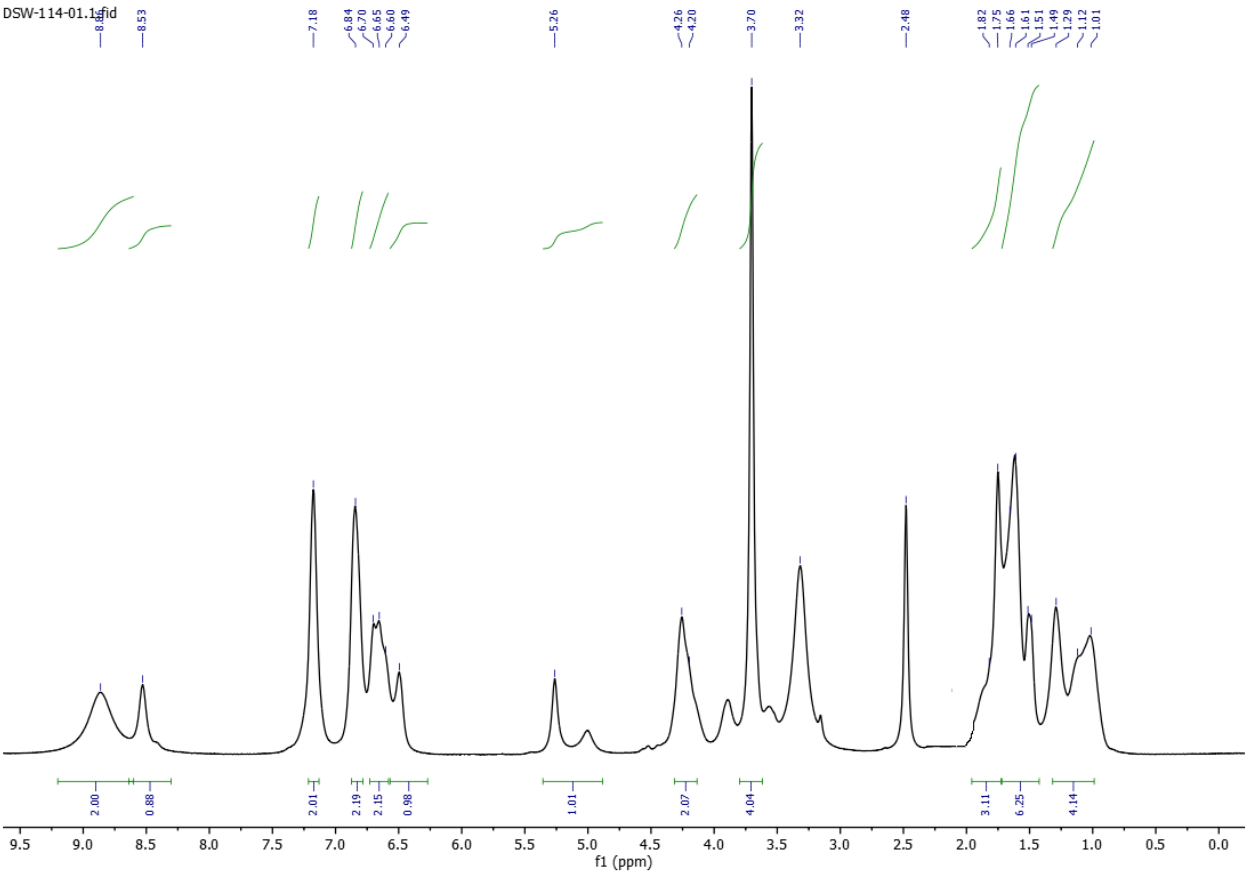


**Figure S27.** $^{1}H$ NMR (400 MHz, DMSO) spectra of compound **5n**

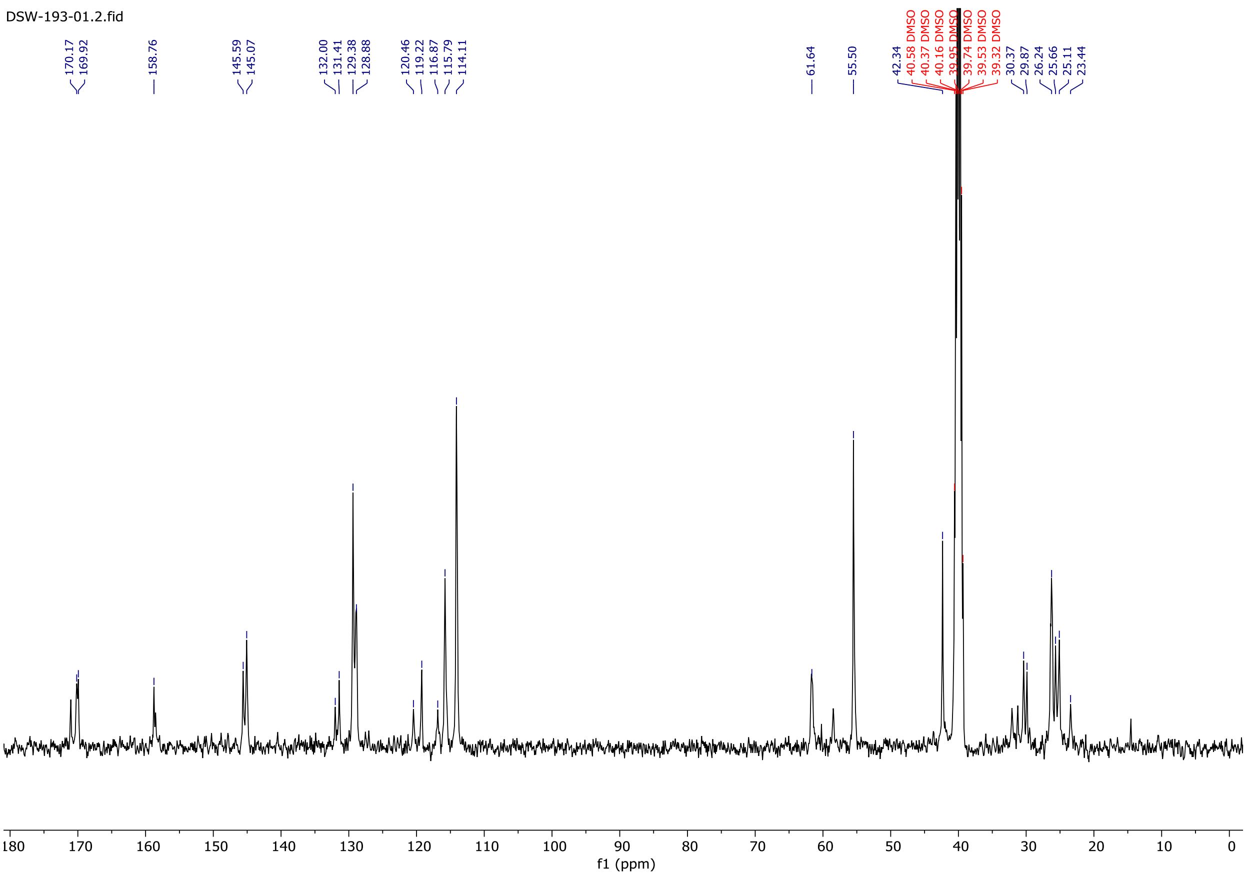


**Figure S28.** $^{13}C$ NMR (400 MHz, DMSO) spectra of compound **5n**

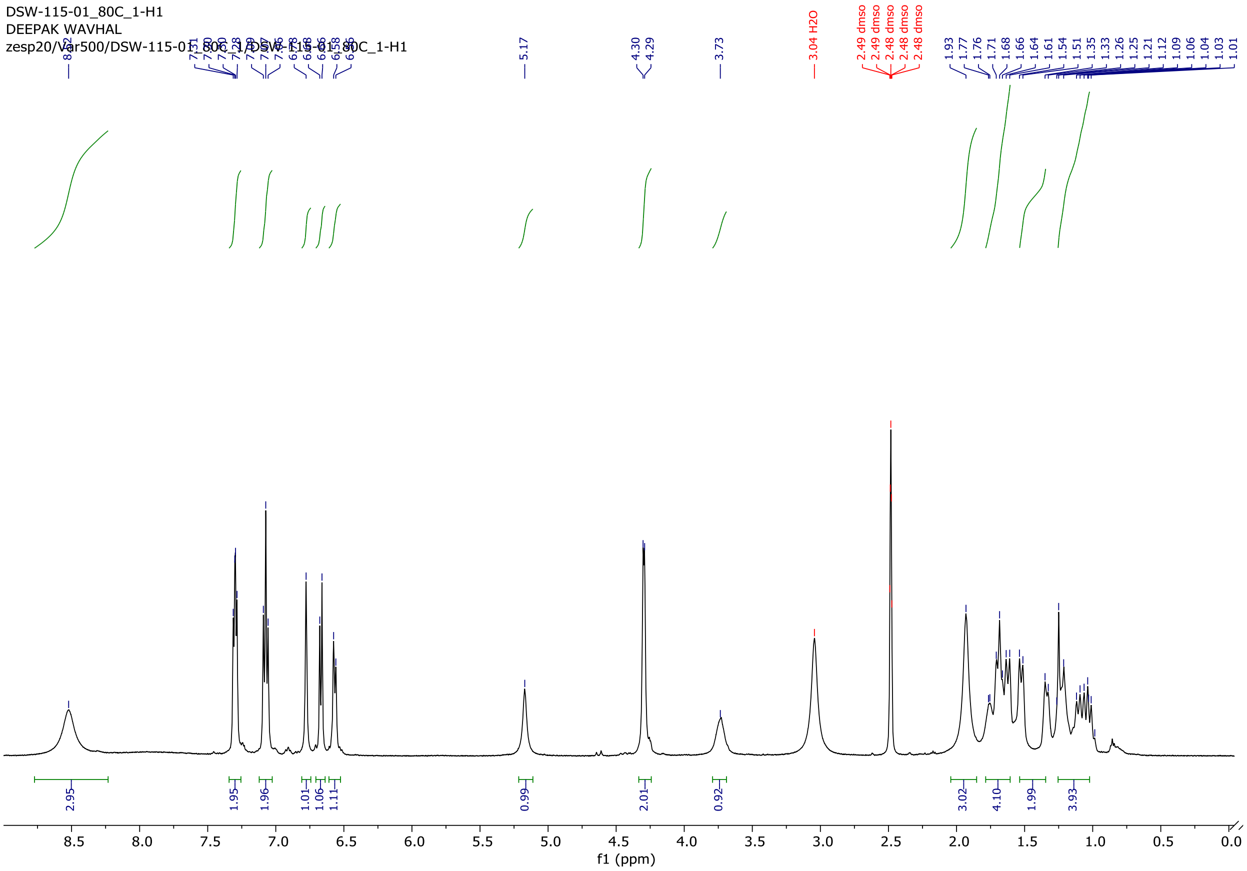


**Figure S29.** $^{1}H$ NMR (400 MHz, DMSO) spectra of compound **5o**

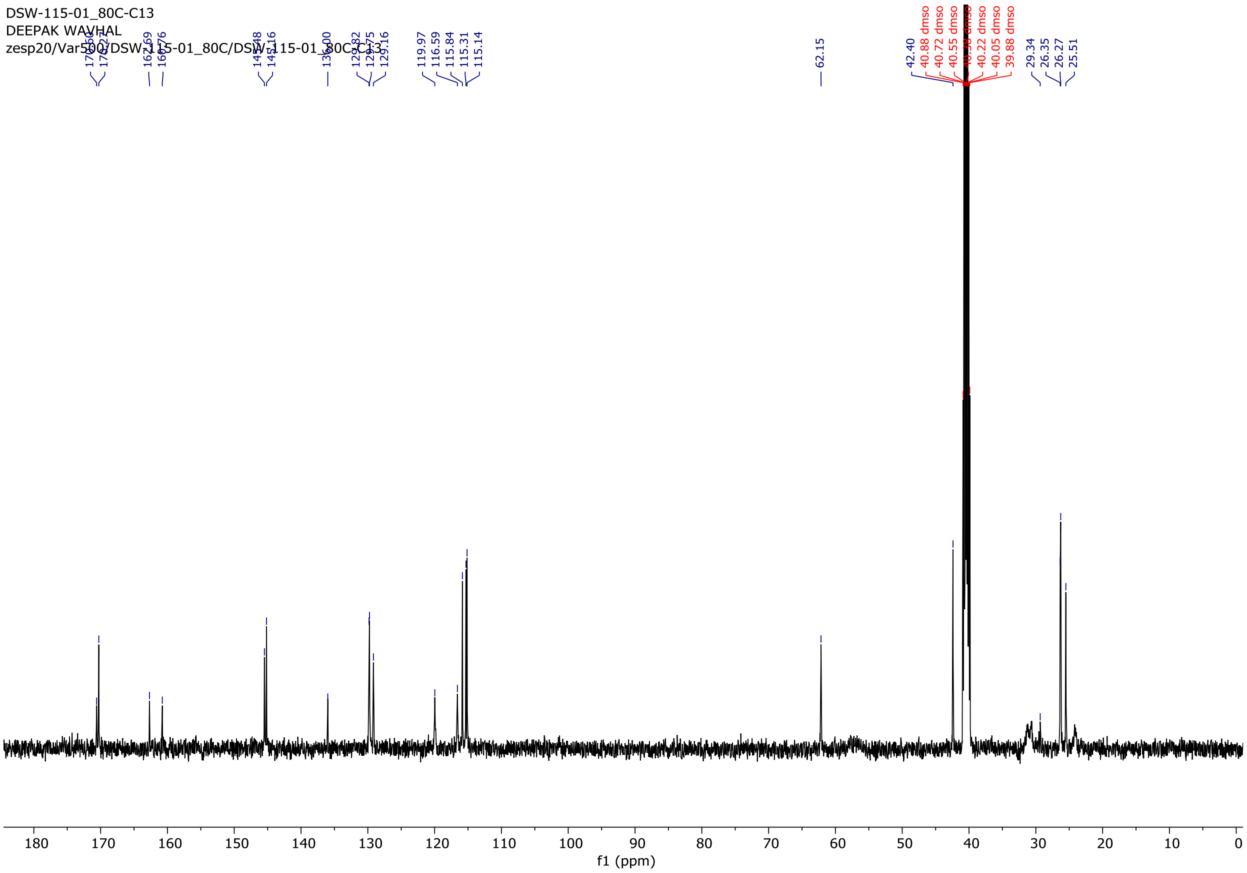

**Figure S30.** $^{13}$C NMR (400 MHz, DMSO) spectra of compound **5o**

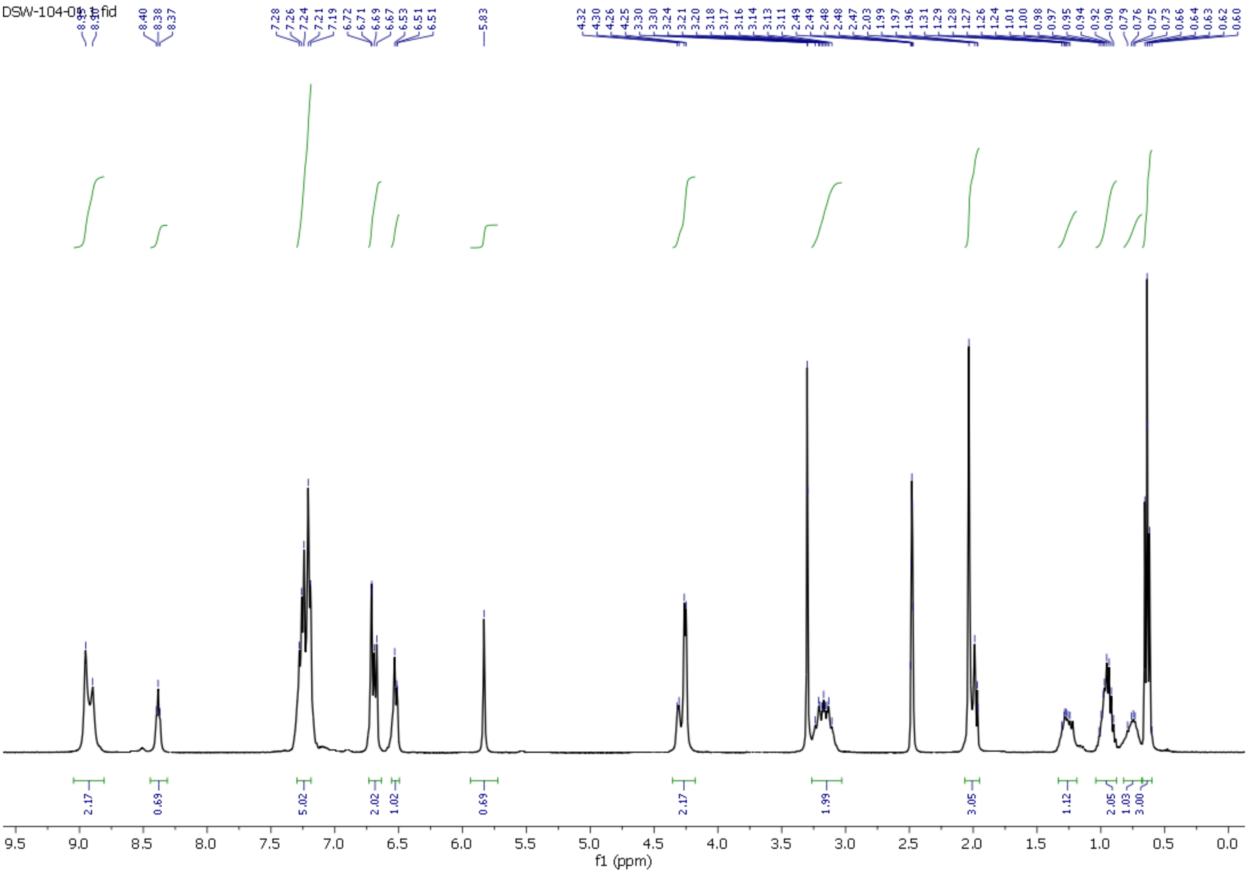

**Figure S31.** $^{1}$H NMR (400 MHz, DMSO) spectra of compound **5p**

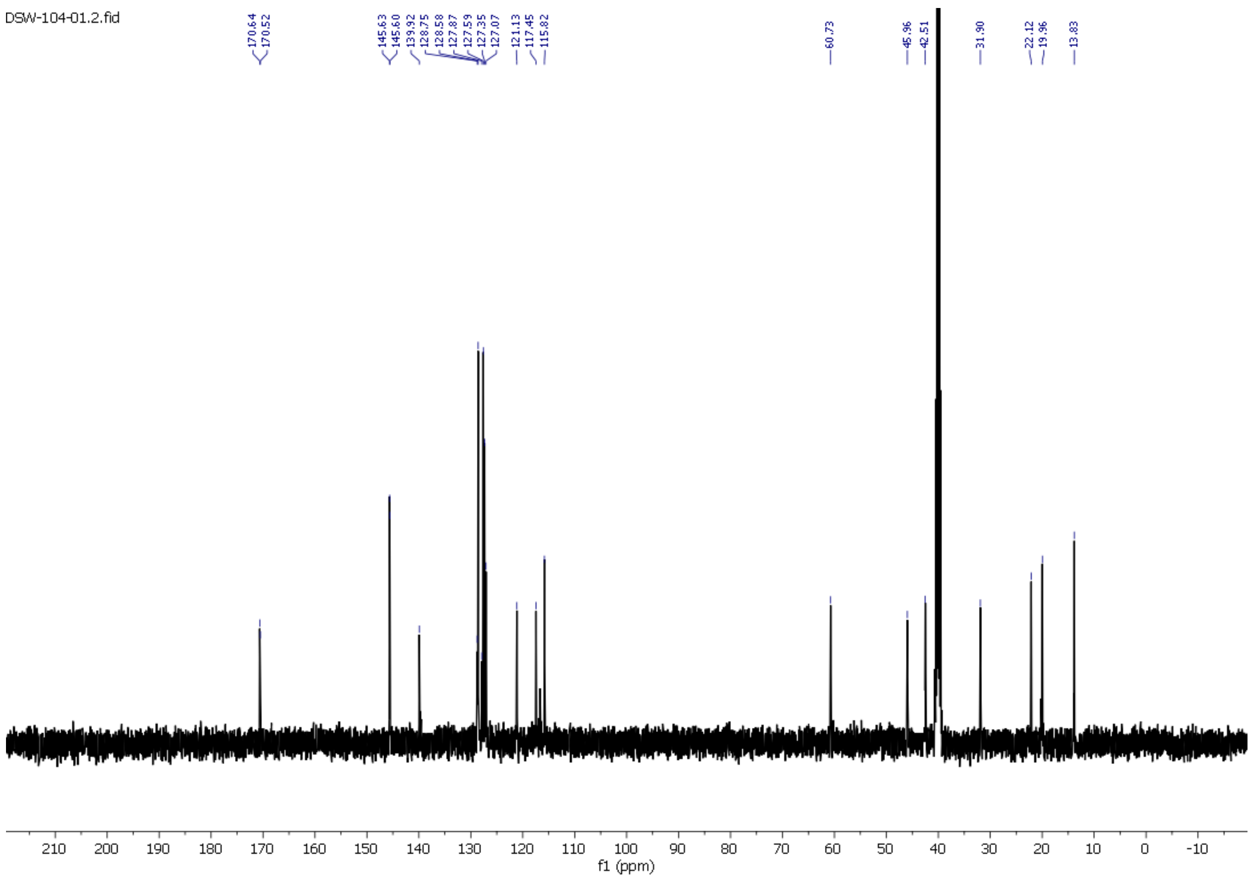

**Figure S32.** $^{13}C$ NMR (100 MHz, DMSO) spectra of compound **5p**

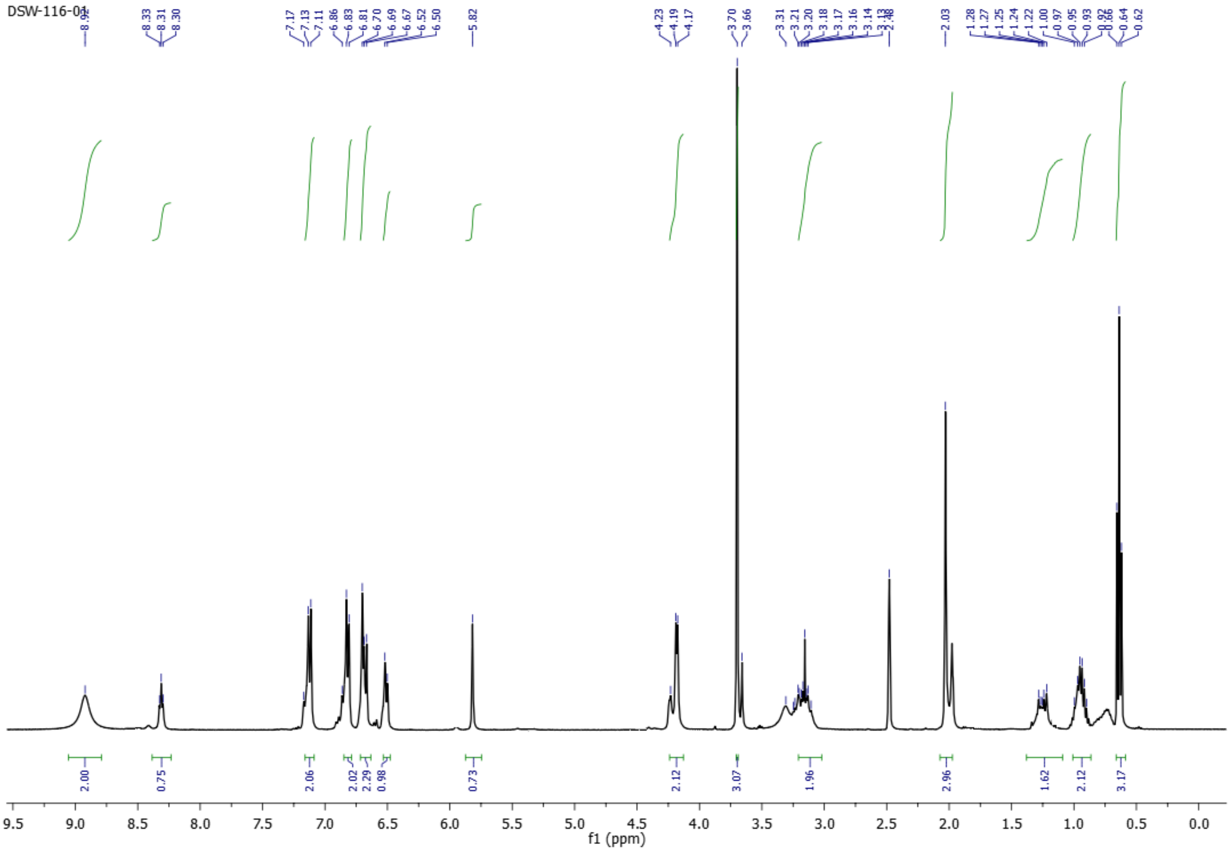

**Figure S33.** $^{1}H$ NMR (400 MHz, DMSO) spectra of compound **5q**

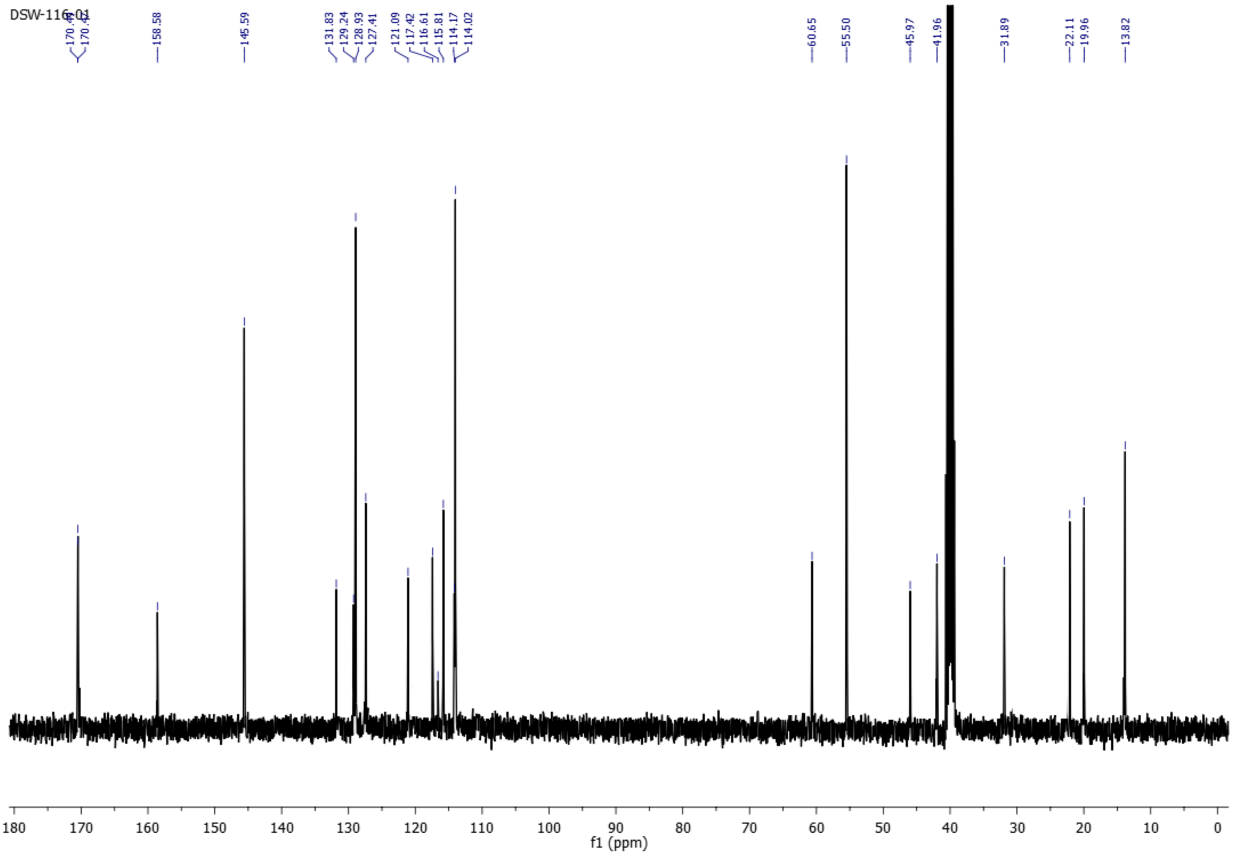

**Figure S34.** $^{13}C$ NMR (100 MHz, DMSO) spectra of compound **5q**

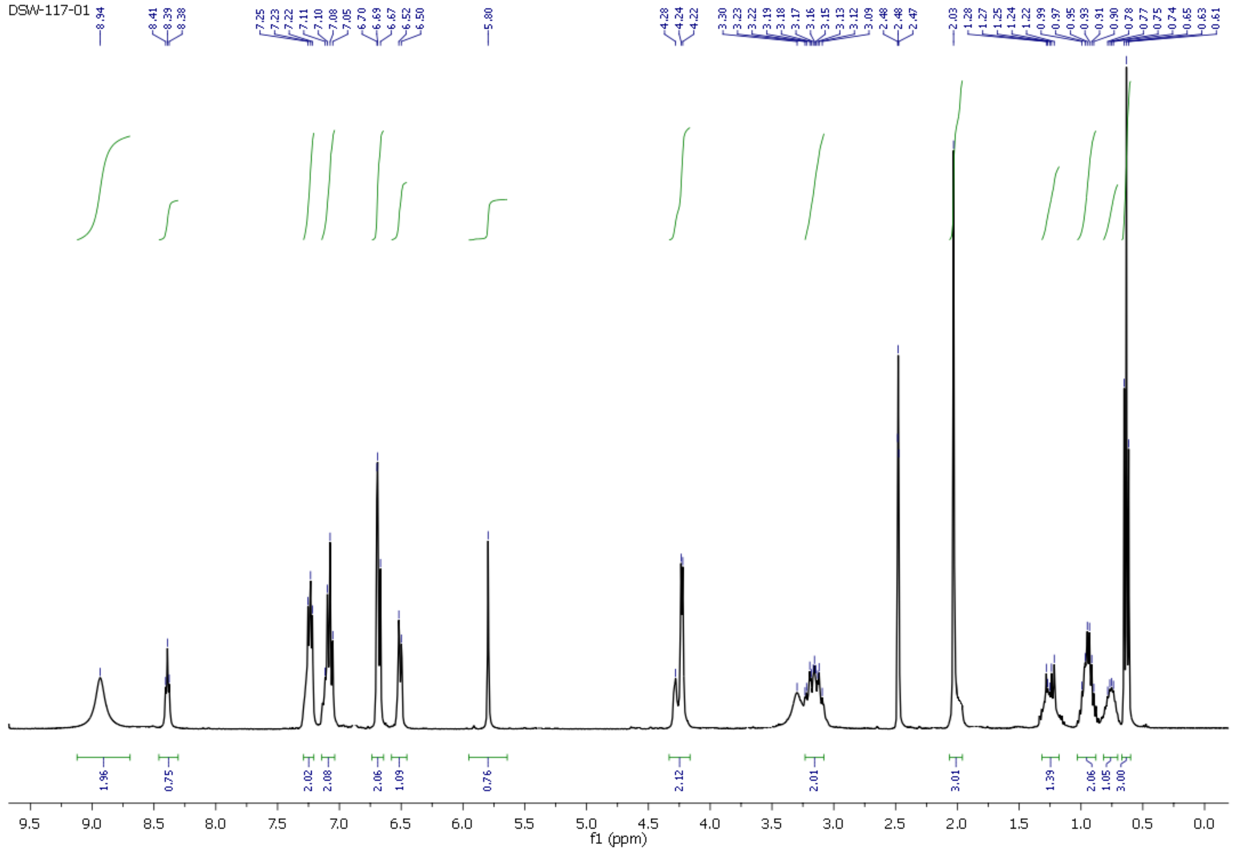

**Figure S35.** $^{1}H$ NMR (400 MHz, DMSO) spectra of compound **5r**

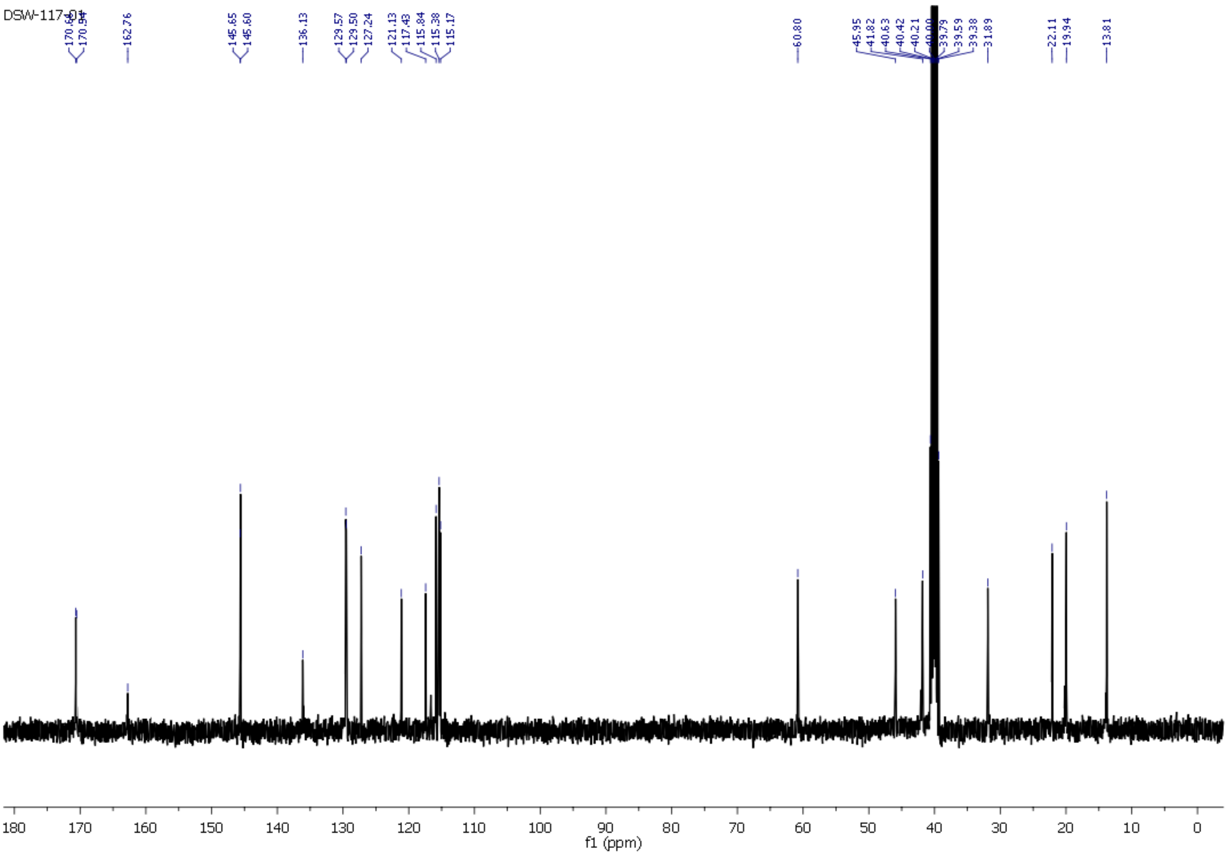


**Figure S36.** $^{13}C$ NMR (100 MHz, DMSO) spectra of compound **5r**

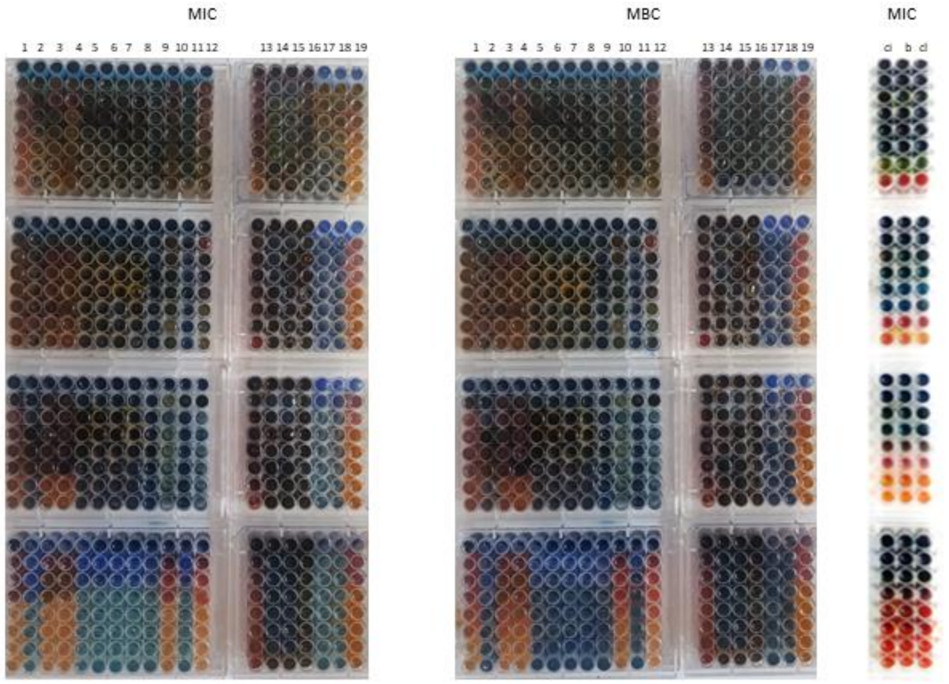


**Figure S37.** Examples of MIC and MBC on microplates with different concentration of studied compounds (µg/mL$^{-1}$). Resazurin was added as an indicator of microbial growth with *E. coli* K12, R2, R3, and R4 strains with tested compounds. Additionally, examples of MIC with different strains K12, R2, R3, and R4 of studied antibiotics with ciprofloxacin (ci), bleomycin (b), and cloxacillin (cl) in (µg/mL).

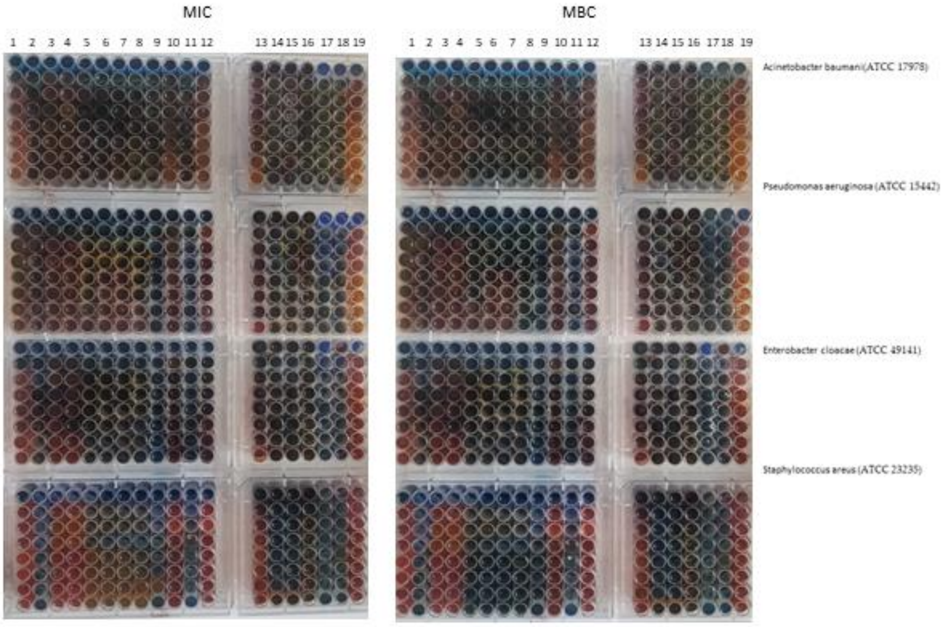


**Figure S38.** Examples of MIC and MBC on microplates with different concentration of studied compounds (µg/mL). Resazurin was added as an indicator of microbial growth with *Staphylococcus aureus strain* (ATCC 23235), as well as on *Acinetobacter baumannii* (ATCC 17978), *Pseudomonas aeruginosa* (ATCC 15442), *Enterobacter cloacae* (ATCC 49141) strains with tested compounds.

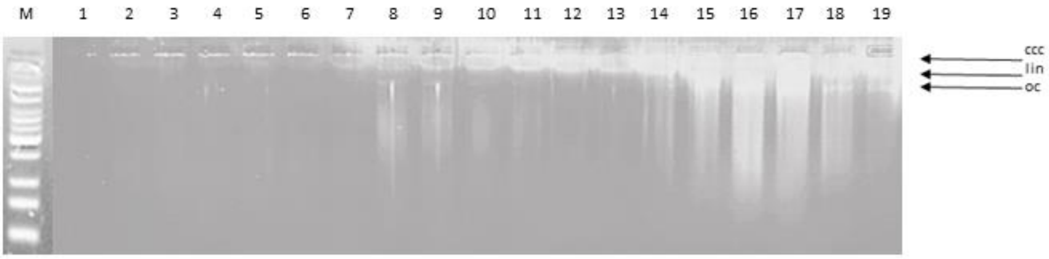


**Figure S39.** An example of an agarose gel electrophoresis separation of isolated plasmids DNA on R4 strains modified with selected peptidomimetic derivatives (lanes 1-19), and digested with repair Fpg protein. M = marker.

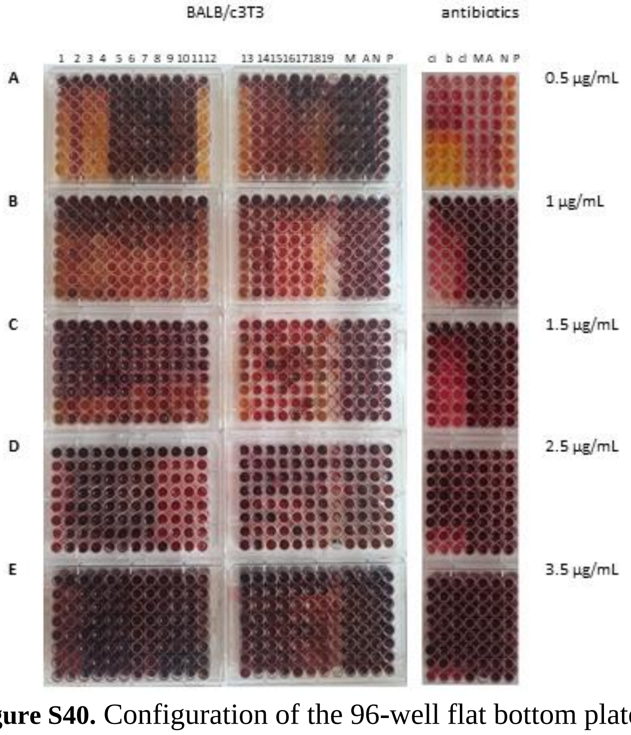


**Figure S40.** Configuration of the 96-well flat bottom plates in which the cytotoxicity assay was carried

out on mouse embryonic fibroblast cell line BALB/c3T3 cells **.** M= complete medium without cells, A= complete medium without extract solutions. N=negative control P=positive control.
Panel A- 0.5 µg/mL concentration, Panel B - 1 µg/mL concentration, Panel C- 1.5 µg/mL concentration, Panel D – 2.5 µg/mL concentration, Panel E- 3.5 µg/mL concentration. A= complete medium without extract solutions. N=negative control P=positive control. Lanes from 1-19 tested compounds . Lanes ci-ciprofloxacin, bl-bleomycin , cl- cloxacillin.

**Table S1:** MIC and MBC values of the synthesized compounds with their SEM deviations.

| Strains/compound | **1** | **5a** | **5b** | **5c** | **5d** | **5e** | **5f** | **5g** | **5h** | **5i** | **5j** | **5k** | **5l** | **5m** | **5n** | **5o** | **5p** | **5q** | **5r** |
|---|---|---|---|---|---|---|---|---|---|---|---|---|---|---|---|---|---|---|---|
| | MIC (µM) and (± Standard Error of the Mean (SEM)) | | | | | | | | | | | | | | | | | | |
| K12 | 1.39±0.05 | 0.30±0.064 | 0.45±0.018 | 0.45±0.018 | 0.36±0.01 | 0.3±0.01 | 0.22±0.01 | 0.42±0.16 | 0.25±0.15 | 0.6±0.03 | 0.26±0.01 | 0.58±0.02 | 0.64±0.03 | 0.35+0.01 | 0.39±0.01 | 0.30±0.06 | 0.33±0.01 | 0.43±0.02 | 0.74±0.03 |
| R2 | 2.46±0.01 | 0.59±0.023 | 0.98±0.04 | 1.0±0.04 | 0.68±0.03 | 0.71±0.03 | 0.65±0.02 | 0.94±0.03 | 0.86±0.03 | 1.17±0.05 | 0.74±0.03 | 1.27±0.06 | 1.17±0.04 | 0.94±0.04 | 0.76±0.03 | 0.79±0.03 | 0.84±0.03 | 0.82±0.03 | 1.50±0.06 |
| R3 | 2.85±0.19 | 0.66±0.026 | 1.61±0.064 | 1.61±0.06 | 0.92±0.04 | 0.89±0.34 | 0.83±0.03 | 1.02±0.04 | 1.07±0.05 | 1.92±0.07 | 1.08±0.04 | 2.13±0.08 | 1.64±0.06 | 1.24±0.04 | 1.10±0.04 | 1.01±0.04 | 1.27±0.05 | 1.13±0.04 | 2.56±0.10 |
| R4 | 3.57±0.65 | 0.88±0.35 | 2.0±0.08 | 1.98±0.08 | 1.45±0.06 | 1.37±0.05 | 1.28±0.05 | 1.5±0.06 | 1.36±0.05 | 2.31±0.01 | 1.22±0.05 | 2.55±0.10 | 2.61±0.10 | 0.59±0.06 | 1.48±0.06 | 1.52±0.06 | 1.58±0.06 | 1.49±0.06 | 3.13±0.12 |
| Acinetobacter baumannii | 2.82±0.18 | 0.63±0.025 | 0.83±0.03 | 0.84±0.33 | 0.74±0.03 | 0.72±0.03 | 0.68±0.02 | 0.79±0.02 | 0.73±0.03 | 0.99±0.04 | 0.80±0.02 | 0.93±0.03 | 1.00±0.04 | 0.79±0.03 | 0.75±0.03 | 0.75±0.03 | 0.87±0.03 | 0.81±0.03 | 1.26±0.05 |
| Pseudomonas aeruginosa | 3.0±0.55 | 0.62±0.024 | 0.96±0.04 | 1.04±0.04 | 0.63±0.02 | 0.67±0.02 | 0.64±0.02 | 0.70±0.02 | 0.69±0.02 | 1.23±0.05 | 0.77±0.03 | 1.15±0.04 | 1.23±0.06 | 0.80±0.03 | 0.78±0.03 | 0.73±0.03 | 0.88±0.03 | 0.83±0.03 | 1.64±0.06 |
| Enterobacter | 2.75±0.2 | 0.58±0.023 | 1.0±0.04 | 1.08±0.04 | 0.68±0.03 | 0.64±0.02 | 0.68±0.02 | 0.72±0.02 | 0.74±0.02 | 1.21±0.05 | 0.76±0.03 | 1.14±0.04 | 1.23±0.06 | 0.81±0.03 | 0.80±0.03 | 0.80±0.03 | 0.86±0.03 | 0.84±0.03 | 1.67±0.06 |
| Staphylococcus areus | 2.67±0.17 | 0.65±0.026 | 0.90±0.03 | 0.96±0.04 | 0.68±0.03 | 0.67±0.02 | 0.68±0.02 | 0.73±0.02 | 0.67±0.02 | 0.99±0.04 | 0.78±0.03 | 1.18±0.05 | 1.27±0.06 | 0.75±0.03 | 0.72+0.03 | 0.74±0.03 | 0.87±0.03 | 0.81±0.03 | 1.51±0.06 |
| | MBC (µM) and (± Standard Error of the Mean (SEM)) | | | | | | | | | | | | | | | | | | |
| K12 | 1.82±0.07 | 0.32±0.01 | 0.61±0.02 | 0.66±0.03 | 0.74±0.03 | 0.87±0.35 | 0.61±0.02 | 0.71±0.03 | 0.71±0.03 | 1.05±0.04 | 0.82±0.03 | 0.79±0.03 | 0.77±0.03 | 0.85±0.03 | 0.77±0.03 | 0.86±0.03 | 0.89±0.03 | 0.79±0.03 | 0.94±0.04 |
| R2 | 2.60±0.10 | 0.62±0.02 | 1.03±0.04 | 1.04±0.04 | 1.07±0.04 | 0.89±0.35 | 0.75±0.03 | 1.23±0.05 | 1.07±0.04 | 1.16±0.05 | 1.19±0.04 | 1.27±0.05 | 1.21±0.05 | 1.31±0.05 | 1.30±0.05 | 1.23±0.05 | 1.51±0.06 | 1.44±0.06 | 1.51±0.06 |
| R3 | 2.96±0.12 | 0.84±0.03 | 1.70±0.07 | 1.79±0.07 | 1.06±0.04 | 1.15±0.05 | 1.18±0.04 | 1.23±0.05 | 1.19±0.04 | 2.13±0.08 | 1.28±0.05 | 2.58±0.10 | 2.62±0.10 | 1.38±0.06 | 1.30±0.05 | 1.45±0.06 | 1.64±0.06 | 1.52±0.06 | 3.14±0.12 |
| R4 | 3.58±0.14 | 1.15±0.04 | 2.21±0.09 | 2.36±0.09 | 1.29±0.05 | 1.29±0.05 | 1.30±0.05 | 1.41±0.06 | 1.42±0.06 | 2.51±0.10 | 1.47±0.06 | 2.82±0.11 | 2.76±0.11 | 1.53±0.06 | 1.44±0.06 | 1.47±0.06 | 1.70±0.07 | 1.58±0.06 | 3.50±0.14 |
| Acinetobacter baumannii | 3.55±0.14 | 0.68±0.02 | 0.90±0.04 | 0.94±0.04 | 0.75±0.03 | 0.75±0.03 | 0.71±0.03 | 0.82±0.33 | 0.75±0.03 | 1.05±0.04 | 0.85±0.03 | 1.00±0.04 | 1.03±0.04 | 0.88±0.03 | 0.79±0.03 | 0.81±0.03 | 0.90±0.04 | 0.85±0.03 | 1.31±0.05 |
| Pseudomonas aeruginosa | 3.33±0.13 | 0.66±0.03 | 0.99±0.04 | 1.09±0.04 | 0.74±0.03 | 0.69±0.03 | 0.69±0.03 | 0.78±0.03 | 0.72±0.03 | 1.26±0.05 | 0.82±0.03 | 1.21±0.05 | 1.26±0.05 | 0.91±0.04 | 0.82±0.03 | 0.86±0.03 | 0.94±0.04 | 0.87±0.03 | 1.49±0.06 |
| Enterobacter | 3.04±0.12 | 0.62±0.02 | 1.05±0.04 | 1.11±0.04 | 0.76±0.03 | 0.70±0.03 | 0.73±0.03 | 0.77±0.03 | 0.73±0.03 | 1.27±0.05 | 0.81±0.03 | 1.26±0.05 | 1.27±0.05 | 0.89±0.03 | 0.80±0.03 | 0.84±0.03 | 0.94±0.04 | 0.85±0.03 | 1.50±0.06 |
| Staphylococcus areus | 3.33±0.13 | 0.68±0.02 | 0.96±0.04 | 1.00±0.04 | 0.74±0.03 | 0.69±0.02 | 0.71±0.03 | 0.80±0.03 | 0.67±0.03 | 1.05±0.05 | 0.74±0.03 | 1.18±0.05 | 1.28±0.05 | 0.78±0.03 | 0.83±0.03 | 0.81±0.03 | 0.94±0.04 | 0.91±0.04 | 1.54±0.06 |
